# Soil organic carbon in agricultural soils of an inter-Andean valley in Colombia: understanding the effects of environmental and geographic variables

**DOI:** 10.1007/s10661-025-14123-1

**Published:** 2025-05-30

**Authors:** Andrea Hoyos-Sanclemente, Juan Carlos Menjivar-Flores, German Rueda-Saa

**Affiliations:** 1 Departamento de Ingeniería, Facultad de Ingeniería y Administración, Universidad Nacional de Colombia, Sede Palmira, Palmira, Colombia; 2 Departamento de Ciencias Agrícolas, Facultad de Ciencias Agropecuarias, Universidad Nacional de Colombia, Sede Palmira, Palmira, Colombia

**Keywords:** Agricultural soils, Kriging methods, Soil order, Soil organic carbon, Spatial variability

## Abstract

This study assessed soil organic carbon (SOC) content and its spatial variability at 130 agricultural sites of an inter-Andean valley in the Guachal watershed in southwestern Colombia. The research tested two hypotheses: (1) the SOC content varies significantly across orders, types, and environmental conditions and (2) geostatistical methods incorporating auxiliary variables improve the accuracy of SOC prediction. Ordinary kriging (OK) and cokriging (CK) techniques were used to map SOC based on soil properties and environmental variables. The content of SOC varied from 20 to 294 t ha^−1^. The CK performed better than OK by including additional variables such as total nitrogen, pH, and Fe, reducing prediction error and improving spatial accuracy. The highest SOC content (174.83 t ha^−1^) was observed in soils from the order andisols located on steep slopes (50–75%) and in cold climates (12–18 °C) with secondary vegetation. Soils from the order mollisol, located in flat areas, with temperatures above 24 °C and covered by cultivated pastures, had the lowest organic carbon content (20.42 t ha^−1^). The major agricultural crops (sugarcane and cocoa) showed minimal variability in SOC reserves. These results indicate the critical role of soil order, land use, and environmental factors in SOC distribution. Furthermore, they provide essential information for policies aimed at improving carbon sequestration and sustainable soil management in tropical agricultural landscapes.

**Supplementary information:**

The online version contains supplementary material available at 10.1007/s10661-025-14123-1.

## Introduction

The soil contains significant amounts of carbon that exceed the stocks of the atmosphere and terrestrial vegetation combined (Li et al., 2024; Potash et al., 2023; Rodrigues et al., 2023). However, these are among the most vulnerable resources to the impacts of climate change and declines in global biodiversity (IPCC, 2020). These changes are a worldwide concern, as they have unfavorable consequences both for the economy and for people’s quality of life (World Economic Forum, 2024).

Understanding and monitoring organic carbon stocks in the soil environment are indicators of the environmental quality of ecosystems (Bouasria et al., 2020; J. Chen et al., 2023; Fohrafellner et al., 2023). Global estimates of organic carbon (OC) have been carried out, but they vary due to the methodologies used (Bouasria et al., 2022; LIN et al., 2023; Panagos et al., 2024).

The mapping of soil organic carbon (SOC) has been reported in various studies, establishing a correlation between SOC content and various soil factors, including topography, climate, and physicochemical and biological characteristics. Zhou et al. (2023) analyzed the influence of topographic factors on the spatial distribution of SOC in subtropical forests in China. In Nepal, mapping of SOC stocks was carried out utilizing national field data to provide an accurate assessment of forest resources. Additionally, Khanal et al. (2023) emphasized the importance of factors such as topography and vegetation cover for SOC variation.

The stability of SOC is attributed to its resistance to various agricultural management methods, as well as its protection against microbial biodegradation. This is due in part to the influence of clay particles, which effectively adsorb carbon and shield it from enzymatic activity, facilitating humification (Allen et al., 2010). The most persistent organic fractions in the soil decompose slowly and require many years to perceive significant changes. In contrast, the labile fractions of SOC are more sensitive to modifications resulting from agricultural practices such as crop rotation, fertilization, and tillage systems (Bossolani et al., 2021). According to Loayza et al. (2020), SOC storage capacity can be divided into four categories (t ha^−1^): low (< 40.0), medium (40.0 to 79.9), high (80.0 to 119.9), and very high (≥ 120.0).

Different methods have been employed to generate predictive SOC content maps, and OK and CK have consistently demonstrated superiority in many applications. These geostatistical techniques take advantage of the spatial autocorrelation of soil data, providing more accurate and reliable estimates than traditional interpolation methods such as random forests, neural networks, and inverse distance-weighted (IDW) interpolation (Bouasria et al., 2021). According to Chen et al. (2019), the efficacy of OK and CK frequently surpasses that of other methods in terms of precision and accuracy. They are capable of capturing complex spatial patterns with limited data availability (Hengl et al., 2014; Kahraman et al., 2024). Furthermore, even in studies where random forests and neural networks have demonstrated effectiveness, OK and CK remain valuable tools for understanding and predicting SOC distribution in environmental contexts (Farooq et al., 2022; Szatmári & Pásztor, 2019).

Other authors have developed studies on SOC mapping and analysis. The study by Chabala et al. (2017) focused on the application of ordinary kriging in an area with 100 monitoring sites. Funes et al. (2019) estimated SOC stocks using soil profiles and ArcGIS to map their variability. The authors also considered environmental factors, agricultural practices, and soil properties. Recently, Zhu et al. (2022) addressed the prediction of SOC content in soil samples in Jurong, China, by employing machine learning and radial kriging.

Rainford et al. (2021) estimated SOC stocks in the Colombian Eastern Plains using a random forest model and nine layers of environmental covariates. Bolívar Gamboa et al. (2021) estimated the content and distribution of OC throughout the country using regression kriging for predictive spatial modeling. The emphasis was on the estimation of SOC and its distribution up to a depth of 30 cm.

The national and international interest in the study of SOC content is closely linked to the mitigation of climate change and the sustainability of agriculture in Colombia, which has wide biological and geographic diversity. Other studies have emphasized the significance of soil carbon management, not only for sequestration but also for enhancing soil resilience to climate change. The crucial connection between soil management practices and ecosystem sustainability should be recognized (Baveye et al., 2020). Furthermore, Colombia plays a crucial role in international climate agreements, such as the Paris Agreement. A commitment to soil carbon sequestration is essential for controlling the increase in CO2 emissions in this area.

Therefore, soil quality monitoring and assessment in agricultural areas of the country and knowledge of SOC stocks are considered relevant studies. This research aimed to analyze the SOC content and distribution in agricultural soils of an inter-Andean valley in the Guachal watershed in southwestern Colombia. Different environmental and geographic conditions such as soil cover, slope, microclimate, soil texture, and soil order were analyzed to determine their influence on COS variability. Furthermore, a predictive mapping of COS was performed using ordinary kriging (OK) and cokriging (CK) geostatistical techniques to improve the spatial accuracy of the estimates.

## Materials and methods

### Study area and sampling sites

**Fig. 1:**
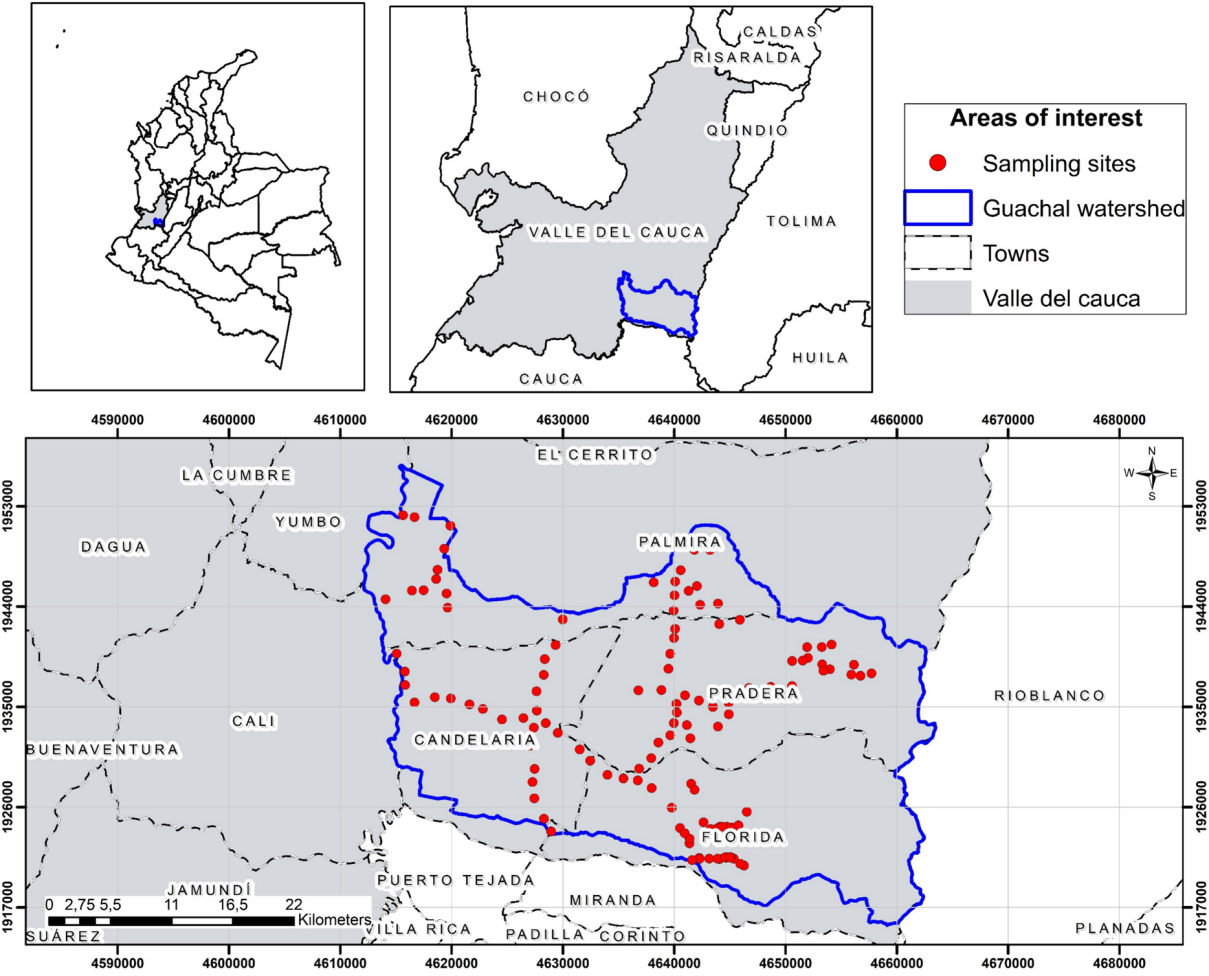
Study area and distribution of sampling sites in the Guachal watershed, Valle del Cauca, Colombia

The study was conducted in the soils of the Guachal watershed, located in the inter-Andean valley of the Cauca River between the western and central mountain ranges in southwestern Colombia. The study area is located between latitude north 3°13′49″ and longitude west 76°01′47″, with an estimated area of 116,248.28 ha (Fig. 1). The altitude ranges from 940 to 3800 m above sea level, which contributes to the high variability in humidity and temperature conditions across the landscape (Espinosa & Muñoz, 2015). According to the climatic zoning of Caldas-Lang adapted by IDEAM (2017), the region comprises various climatic types, including warm semiarid, warm semihumid, temperate semihumid, cold humid, and very cold superhumid zones. This classification was used because it provides detailed zoning tailored to Colombian territory and integrates both thermal floors and precipitation regimes, making it particularly suitable for ecological and soil-related studies in tropical mountainous environments (Medina & Aldana, 2019).

The Guachal watershed has a long history of intensive agricultural use, dominated by sugarcane cultivation, which began expanding significantly in the mid-twentieth century (Uribe Castro, 2014). Pastures and other permanent crops, such as cocoa, also contribute to the region’s land use (Cadavid et al., 2019; Garcia Gonzalez et al., 2018). Traditional agricultural practices, such as conventional tillage, monoculture, and limited application of conservation techniques, have influenced carbon stocks, accelerated the mineralization of organic matter, and reduced SOC retention capacity (Camacho et al., 2021; Gómez-Balanta & Ramírez-Náder, 2022). In recent decades, there has been a gradual incorporation of sustainable practices, such as crop rotation and residue management, although these are not yet widely adopted (Ramírez et al., 2021). The study area represents a combination of traditional and sustainable land management practices, providing a unique context for analyzing SOC variability.

A cartographic analysis was performed using ArcGIS Pro software to define the study area and sampling sites. The sampling areas were determined by considering main, secondary, and tertiary access roads, and applying buffers of 1 km, 500 m, and 250 m, respectively. The points were uniformly distributed within the buffers, guaranteeing vehicular accessibility at each site. Soil order and cover type were used to classify sites, and as environmental geography to assess their influence on soil organic carbon content (COS). Weather conditions, accessibility, terrain management, and safety were assessed in situ. If a site was not suitable, the point was relocated to a similar area to maintain spatial proportionality within the watershed.

A total of 130 sampling points were taken from a depth of 0–30 cm. Among the total number of sites evaluated, 63 (48.46%) corresponded to the mollisol order (suborder ustolls predominates), which is predominant in the area, followed by 22 (16.92%) in the Entisol order (suborder orthents predominates) and 15 (11.54%) in the Inceptisol order (suborder ustepts predominates). The sampling database used for modeling includes soil order, suborder, and great group (supplementary material Table A1).

In relation to land use, the soil cover classification and cartographic information of the Corporación Autónoma Regional del Valle del Cauca (CVC) at a scale of 1:25,000 from the year 2023 were used. Sugarcane cultivation dominated at 52,601.97 ha, followed by cultivated pasture (22,204.59 ha) and mixed natural forest (14,814.71 ha).

### Physicochemical properties of the soil, environmental, and geographic variables

The analysis of 22 soil properties was carried out (Table 1). The physicochemical properties of the soil were studied at the Agrosavia Laboratory, accredited for the analysis of the soil matrix. To categorize the values obtained for each physicochemical parameter, the guidelines of Technical Assistance Manual No. 25 of the Colombian Agricultural Institute were used. This manual is an essential reference for establishing standards and criteria for agricultural research in Colombia. To identify and recognize the soil characteristics, an exploratory analysis of the physicochemical characteristics of the soils in the Guachal watershed was carried out. The analysis included descriptive statistics and an analysis of normality using the Kolmogorov–Smirnov test, where values with a *p* > 0.05 were considered to indicate a normal distribution.

**Table 1:** Soil physicochemical parameters

Parameter	Units	Methods
% sand (% S)	%	Bouyoucos method
% clay (% C)	%	
% silt (% Si)	%	
Textural class	N/A	
Bulk density	g cm^−3^	Metal cylinder method
pH (1:2.5)	Units	GA-R-46, version 05 of 2019–10-02
Electrical conductivity (EC) (1:5)	dS	NTC 5596:2008. Measurement in suspension soil/water ratio 1:5 (weight/volume)
Organic matter (OM)	g 100 g^−1^	NTC 5403 Walkley & Black
Organic carbon (OC)	g 100 g^−1^	GA-R-119 version 2 2019–09–20
Total nitrogen (TN)	g 100 g^−1^	Kjeldahl
Sulfur (S)	mg kg^−1^	Monobasic calcium phosphate
Boron (B)	mg kg^−1^	
Phosphorus (P) (Bray II)	mg kg^−1^	GA-R-48, version 05 of 2019–10-02
Calcium (Ca)	cmol(+) kg^−1^	
Magnesium (Mg)	cmol(+) kg^−1^	
Potassium (K)	cmol(+) kg^−1^	
Sodium (Na)	cmol(+) kg^−1^	
Iron (Fe) Olsen	mg kg^−1^	NTC 5526:2007
Copper (Cu) Olsen	mg kg^−1^	
Manganese (Mn) Olsen	mg kg^−1^	
Zinc (Zn) Olsen	mg kg^−1^	
Cation exchange capacity (CEC)	cmol(+) kg^−1^	1 N ammonium acetate pH 7.0

All environmental and geographic variables were systematically recorded at each of the 130 sampling points. Soil cover was determined through the following: (1) direct field observation using standardized protocols and (2) validation against the 2023 soil cover map from the CVC (1:25,000 scale). The soil order, cover, slope, microclimate, and texture were all included in the analysis. The correlations between the SOC contents at each sampling site and various environmental variables were examined. For each variable, box plots were constructed to visualize the variability and distribution of SOC across the different categories.

The soil order analysis was performed to assess the relationship between the SOC content and the structural characteristics of the soil, as well as its capacity for water retention. The soil order was categorized based on the USDA soil classification system, and the SOC content for each soil order was compared. This analysis helps to identify how different soil types influence carbon storage capacity, considering their physical properties, such as structure and water retention potential. Furthermore, the influence of vegetation cover, topography, and climatic conditions on soil carbon dynamics was examined. Finally, differences in SOC content between soil textures were analyzed.

### Soil organic carbon stocks

SOC stocks were estimated for the topsoil layer (0 to 30 cm). The estimation technique implemented is recommended by Clara et al. (2017). The SOC content was expressed in tons of carbon stored in the soil (t ha^−1^), *d* is the depth (30 cm), BD corresponds to the bulk density (g/cm^3^), and OC is the organic carbon content (g 100 g^−1^) (Eq. 1).

1 $$\text{SOC}=d \times \text{BD }\times \text{OC}$$

It was decided not to incorporate the adjustment factor for the coarse fraction. The fine fraction of OC was determined directly in the laboratory (< 0.106 mm). Although there is no defined methodology for determining labile organic carbon content in soil (Zhang et al., 2020), using a quantification method for particles smaller than 0.250 mm has proven to be effective in identifying the most stable fractions of stored organic carbon (Sainepo et al., 2018).

### Mapping of soil organic carbon and spatial distribution

To map the soil organic carbon (SOC) content in the study area (Guachal watershed), two advanced spatial interpolation techniques, ordinary kriging (OK) and cokriging (CK), were used. Geostatistical analysis by OK is an advanced spatial interpolation technique used to estimate values at unknown points based on the values of known points. The OK technique takes advantage of spatial autocorrelation, which suggests that nearby points are more similar to those farther away (Ramírez Castañeda, 2023). A logarithmic transformation of the SOC (t ha^−1^) data was executed to ensure that the data met the normality assumptions required for the correct application of ordinary kriging. Using the transformed data, SOC values in the area were predicted, generating a detailed map of the spatial distribution in the Guachal watershed. These mapping procedures involved several general steps, such as data exploration, semivariogram modeling, and map generation and validation, following standard practices in spatial analysis (Inocencio-Vasquez & Florida-Rofner, 2022).

The CK improved the accuracy of the predictions by incorporating auxiliary variables that had a high correlation with the SOC. For the present study, physicochemical variables that showed high and moderate correlations (Pearson) with the SOC and were statistically significant (*p* < 0.05) were selected (Mallik et al., 2022). The auxiliary variables were also logarithmically transformed to approximate a normal distribution. This transformation is essential for meeting normality requirements and improving the accuracy of the CK model. Using the transformed auxiliary variables, the CK was used to predict SOC values. The CK considers both the spatial autocorrelation of SOC and the correlation between SOC and auxiliary variables, which allows for an accurate prediction compared to OK (L. Chen et al., 2019). Finally, three detailed maps of the spatial distribution of SOC in the Guachal watershed were generated using OK and CK.

## Results and discussion

### Physicochemical properties of the soil

The results of the univariate analysis of the physicochemical properties of the soil are shown in Table 2. The textural composition (41.78% sand, 27.64% clay, 30.58% silt) directly governs soil hydro-physical dynamics through three key mechanisms: (1) clay particles (< 0.002 mm) provide reactive surfaces for organo-mineral complexation, physically shielding soil organic carbon (SOC) from microbial decomposition (Jin et al., 2024); (2) the sandy fraction (0.05–2 mm) drives pore-size distribution, accounting for the measured bulk density (1.30 g cm^−3^) and rapid drainage characteristics; and (3) the silt/clay ratio (1.11) modulates structural stability, with values > 0.8 typically indicating moderate aggregational resilience (Dewangan, 2023).

**Table 2:** Statistical analysis of the physicochemical parameters

Parameter	Units	Media	Median	Mode	SD	CV	Max	Min	Kurtosis	Skewness
% sand (% S)	%	41.78	42.10	33.93	16.11	38.55	77.50	6.63	− 0.88	− 0.07
% clay (% C)	%	27.64	26.08	55.91	12.61	45.63	61.15	9.08	− 0.60	0.48
% silt (% Si)	%	30.58	30.34	26.40	11.42	37.36	58.26	0.00	0.21	− 0.12
Bulk density	g cm^−3^	1.30	1.37	0.46	0.27	20.67	1.76	0.46	0.57	− 0.87
pH (1:2,5)	units	6.71	6.68	6.67	0.79	11.70	8.86	4.94	0.17	0.38
Electrical conductivity (EC) (1:5)	dS m^−1^	0.39	0.22	0.22	0.91	236.34	10.28	0.10	109.24	10.07
Organic matter (OM)	g 100 g^−1^	3.60	2.73	2.26	2.59	71.82	17.29	0.79	8.73	2.65
Organic carbon (OC)	g 100 g^−1^	2.09	1.57	1.31	1.50	71.91	10.03	0.46	8.72	2.65
Total nitrogen (TN)	g 100 g^−1^	0.23	0.18	0.13	0.15	63.84	0.89	0.08	6.38	2.45
Sulfur (S)	mg kg^−1^	11.74	6.66	4.53	31.92	271.89	359.71	2.05	111.79	10.28
Boron (B)	mg kg^−1^	0.30	0.26	0.26	0.23	74.53	2.01	0.03	24.80	3.76
Phosphorus (P) (Bray II)	mg kg^−1^	43.20	17.07	0.00	122.56	283.69	1042.39	0.00	53.91	7.15
Calcium (Ca)	cmol(+) kg^−1^	16.22	13.49	7.84	9.59	59.11	42.08	2.40	− 0.24	0.86
Magnesium (Mg)	cmol(+) kg^−1^	7.08	5.04	2.69	5.55	78.44	22.72	0.40	− 0.32	0.92
Potassium (K)	cmol(+) kg^−1^	0.48	0.23	0.09	0.76	158.29	5.08	0.09	14.53	3.61
Sodium (Na)	cmol(+) kg^−1^	0.22	0	0	0.74	340.60	7.79	0	86.34	8.80
Iron (Fe) Olsen	mg kg^−1^	87.59	59.80	_	80.58	92.00	413.66	8.68	2.73	1.68
Copper (Cu) Olsen	mg kg^−1^	5.07	4.42	0.00	3.66	72.14	26.52	0.00	8.78	2.11
Manganese (Mn) Olsen	mg kg^−1^	5.35	3.33	0.00	5.43	101.42	39.76	0.00	12.60	2.87
Zinc (Zn) Olsen	mg kg^−1^	1.86	0.00	0.00	3.83	205.73	27.38	0.00	21.55	4.20
Cation exchange capacity (CEC)	cmol(+) kg^−1^	25.45	21.27	_	13.57	53.32	59.21	0.00	− 0.55	0.71
SOC general	t ha^−1^	74.25	62.96	_	41.79	56.28	324.80	20.42	12.26	2.94

*SD*; standard deviation, *CV*; coefficient of variation, *Max*; maximum, *Min*; minimum

**Fig. 2:**
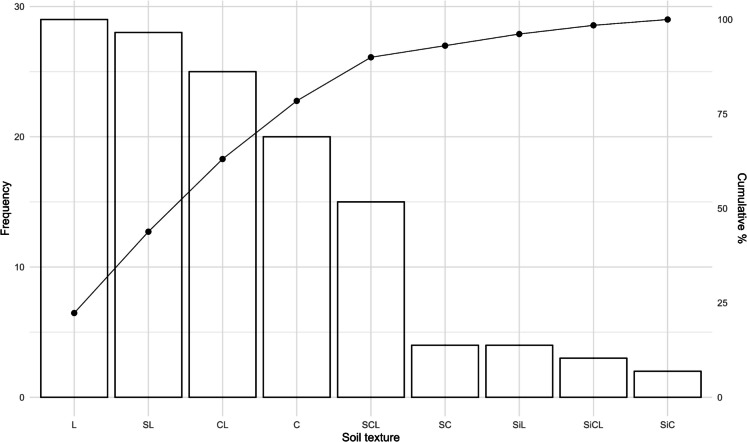
Soil textures in the study area. C, clay; SC, sandy-clay; SiC, silty-clay; L, loam; SL, sandy-loam; CL, clay-loam; SCL, sandy-clay-loam; SiCL, silty-clay-loam; SiL, silty-loam

**Fig. 3:**
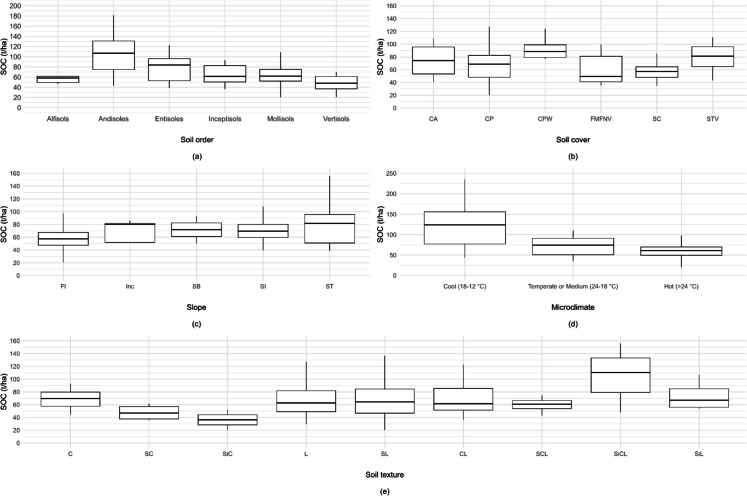
Box plots of environmental and geographic variables and SOC (t ha−1). Note: (**a**) Soil order. (**b**) Soil cover. FMFPC, fragmented mixed forest with pasture and crops; FMFNV, fragmented mixed forest with natural vegetation; CP, cultivated pasture; CPW, cultivated pasture with weeds; CA, cocoa; SC, sugarcane; STV, secondary or transitional vegetation. (**c**) Soil slope. Fl, flat (< 3%); SI, slightly inclined (3–7%); Inc, inclined (7–12%); SI, steeply inclined (12–25%); SB, steeply broken (25–50%); ST, steep (50–75%). (**d**) Microclimate. (**e**) Soil texture. C, clay; SC, sandy-clay; SiC, silty-clay; L, loam; SL, sandy-loam; CL, clay-loam; SCL, sandy-clay-loam; SiCL, silty-clay-loam; SiL, silty-loam

The soil texture demonstrated significant control over organic carbon sequestration patterns (Fig. 2). The predominant texture of loam and sandy loam (44% of samples) correlated with intermediate SOC storage capacity, reflecting balanced moisture retention and aeration properties. The clayey textures showed significant occurrence (15% of samples), exhibiting significantly higher SOC accumulation compared to coarser textures. It is consistent with their enhanced surface reactivity and organo-mineral complexation potential (Lehmann & Kleber, 2015). This stabilization mechanism was partially influenced by the characteristic hydraulic properties of fine-textured soils, which influence microbial activity and decomposition rates (Jin et al., 2024). The observed textural distribution effectively explains the spatial variability in SOC stocks shown in Fig. 3a.

A mean bulk density of 1.30 g cm^−3^ was estimated, which was classified as fine particles (ICA, 1992), with variations ranging from 0.46 g cm^−3^ to a maximum of 1.76 g cm^−3^. These values indicate the variability of soil compaction, which can significantly impact porosity and the availability of water and nutrients to plants (Lu et al., 2021).

The soil pH ranged from 4.94 to 8.86, with an average value of 6.71. According to the Colombian Agricultural Institute (ICA, 1992), this pH is classified as slightly acidic. At the sites evaluated, the pH varied considerably between acidic and alkaline soils. Different studies indicate that pH variability significantly influences nutrient availability and soil biological activity, affecting agricultural management and sustainability (Havlin et al., 2014; Sori et al., 2021).

The mean electrical conductivity (EC) was 0.39 dS m^−1^, with high variability ranging from 0.10 to 10.28 dS m^−1^. Most of the values ranged from no salinity to moderate salinity (ICA, 1992). Areas with the highest EC values, especially those above 8 dS m^−1^, may present limitations for certain crops due to salinity. A recent study confirmed that EC variability can be a crucial indicator of soil properties that directly affect crop growth and agricultural sustainability (Egidijus et al., 2023).

The mean cation exchange capacity (CEC) was 25.45 cmoI(+) kg^−1^, with wide variability ranging from 13.57 to 59.21 cmoI(+) kg^−1^. The results indicate that the CEC is high (ICA, 1992) and that areas with high values may present some limitations for certain crops. This may reflect the possible influence of this variable on soil health and agricultural productivity. CEC is a relevant indicator for sustainable soil management and reflects how different agricultural practices can influence soil quality and thus crop yields (Emamgholizadeh et al., 2023).

A mean organic matter (OM) content of 3.60 g 100 g^−1^, ranging from 0.79 to 8.73 g 100 g^−1^, was found. The OM content in the study area is classified as medium to high (ICA, 1992). The average organic carbon (OC) content was 2.09 g 100 g^−1^, ranging from 0.46 to 10.03 g 100 g^−1^. These values indicate significant heterogeneity in soil composition, which can be attributed to the diverse soil management practices and specific environmental conditions of the region. In addition, they contribute to improving soil structure, increasing water and nutrient holding capacity, and promoting soil biological activity. These are essential aspects for ecosystem health and agricultural productivity (Oldfield et al., 2019).

A detailed evaluation of the chemical properties of soils provides essential information about fertility and management needs in the study area. These values reflect the significant variation in the concentrations of essential nutrients and micronutrients in the soils of this region. According to the classification of the Colombian Agricultural Institute (ICA, 1992), the sulfur (S) content is intermediate, the calcium (Ca) content is high, the magnesium (Mg) content is moderate, the sodium (Na) content is low, the boron (B) content is moderate, the iron (Fe) content is high, the manganese (Mn) content is moderate, the phosphorus (P) content is very high, and the zinc (Zn) content is moderate.

Sulfur is essential for protein and enzyme synthesis in plants since it contributes to the formation of essential sulfur-containing amino acids such as cysteine and methionine (Künstler et al., 2020). The adequate availability of calcium (Ca) and magnesium (Mg) plays a fundamental role in the structural development of plants and in physiological processes such as photosynthesis (González-Villagra et al., 2021). According to Zörb et al. (2014), potassium (K) is important for the regulation of osmoregulation and the activation of enzymes. Iron and zinc have significant influences on functions such as chlorophyll formation, photosynthesis, protein synthesis, and vegetative growth regulation (Krishna et al., 2023).

The total nitrogen values demonstrated remarkable heterogeneity. Nitrogen is an important component of agricultural productivity, as it is essential for plant growth, protein synthesis, and plant structure (Mengel, 2023). Nitrogen availability in the soil increases nutrient use efficiency and improves crop yields (Govindasamy et al., 2023).

### Influence of environmental and geographic variables on soil organic carbon

The relationships between the SOC content and different environmental variables are shown in Fig. 3. This scope includes the effect of soil order and texture on SOC content, slope, and microclimate, as well as soil cover. According to soil order and texture, the andisols had the highest SOC values, 125.71 t ha^−1^ (Fig. 3a), which is related to higher OM contents, mainly due to volcanic activity that characterizes their formation (Calabi-Floody et al., 2015). The textural contrast between clay-rich Andisols and sandy Entisols (Fig. 3a) demonstrates how surface area differences influence organic carbon storage. Smectite clays (600–800 m^2^/g) provide exponentially more stabilization sites than quartz sands (0.01–0.1 m^2^/g) through organo-mineral complexation (Lehmann & Kleber, 2015). The Vertisol soils had lower SOC values (8.07 t ha^−1^), which could be attributed to their dense clay structure, which favors anaerobic decomposition of organic matter (Mohanty et al., 2020). Alfisols are poorly permeable clay soils and have the least variability and lowest SOC values (Ni et al., 2022).

The relationships between the SOC content and the predominant soil cover in the study area are shown in Fig. 3b. Soil cover plays an important role in carbon cycling, influencing the capture and storage of organic carbon, which is fundamental to understanding climate change and environmental sustainability (Dengiz et al., 2015; Edmondson et al., 2014; Wasige et al., 2014).

Fragmented mixed forests with pasture and crops and cultivated grasslands matted had high SOC values, suggesting greater organic matter accumulation due to the protection and stability provided by the vegetation cover. This finding is in accordance with studies that have demonstrated that forested areas can serve as carbon sinks through the fixation of atmospheric CO_2_ and sequestration of CO_2_ in biomass and soil (An et al., 2023; Wang et al., 2024). The soil cover types with the greatest variability were secondary and transitional vegetation, cultivated pasture, cocoa, and fragmented mixed forest with natural vegetation, which had the lowest medians. Secondary or transitional vegetation exhibited great variability and a high median. This cover may reflect an early successional stage in which the soil is still in the process of accumulating organic matter after a disturbance or land use change (Thomas et al., 2020).

The two major crops in the study area are sugarcane and cocoa. Compared with those of the other cover crops, the interquartile range of sugarcane was narrow, which indicates a lower variability in SOC values in these areas. For this type of crop, there were also outliers above the upper whisker with exceptionally high SOC values, which could be associated with specific management practices or local soil and microclimate conditions. Cocoa showed greater variability than did sugarcane or other predominant cover crops in the study area. It is important that strategies are sought in the management of sugarcane crops to improve soil organic matter and optimize carbon sequestration. The adoption of practices such as no-tillage and proper management of crop residues can increase SOC (Cerri et al., 2011). Sustainable management practices can not only improve soil health and crop productivity but also provide environmental benefits through greenhouse gas mitigation.

In terrestrial ecosystems, microclimate and slope are determining factors of carbon dynamics. The slope was determined according to the Geographic Data Dictionary of the National Environmental Licensing Authority (ANLA, 2020). Figure 3c shows that soils with steep slopes displayed greater variability and higher SOC values (CVC, 2023). This difference may be attributable to a reduction in surface erosion and the accumulation of organic matter in natural depressions or terraces. In contrast, flat soils had the lowest SOC values and lower variability. This could be explained by the frequent implementation of intensive agricultural practices in these regions, which resulted in greater mineralization and a decrease in soil organic matter.

These results contrast with the study of Melendez et al. (2020), where the highest SOC contents were found at lower altitudes. The discrepancy in the results could be explained by differences in soil cover, geoforms, and climatic variables between the zones, which directly affect the stability and sequestration of SOC. According to Jiang et al. (2021), the interaction between terrain relief and climatic regimes can significantly influence SOC accumulation patterns, providing an explanation for the variability observed between different studies.

Figure 3d illustrates the relationship between microclimate and SOC levels. Warm climate soils (> 24 °C) have lower levels of SOC than cold climate soils (18–12 °C). Studies have shown that high temperatures promote the mineralization of organic matter, thereby reducing SOC levels (Grzyb et al., 2020). In temperate climates, soils have an intermediate SOC content, which indicates a balance between organic matter accumulation and decomposition.

The soil texture exhibited significant variability in the SOC content (Fig. 3e). It is highlighted that fine textures, as represented by the silty-clay-loam (SiCL) and clay loam (CL) categories, tend to have a higher SOC content, similar to the results reported by Heger et al. (2023).

### Mapping of soil organic carbon and spatial distribution using geostatistical methods

**Fig. 4:**
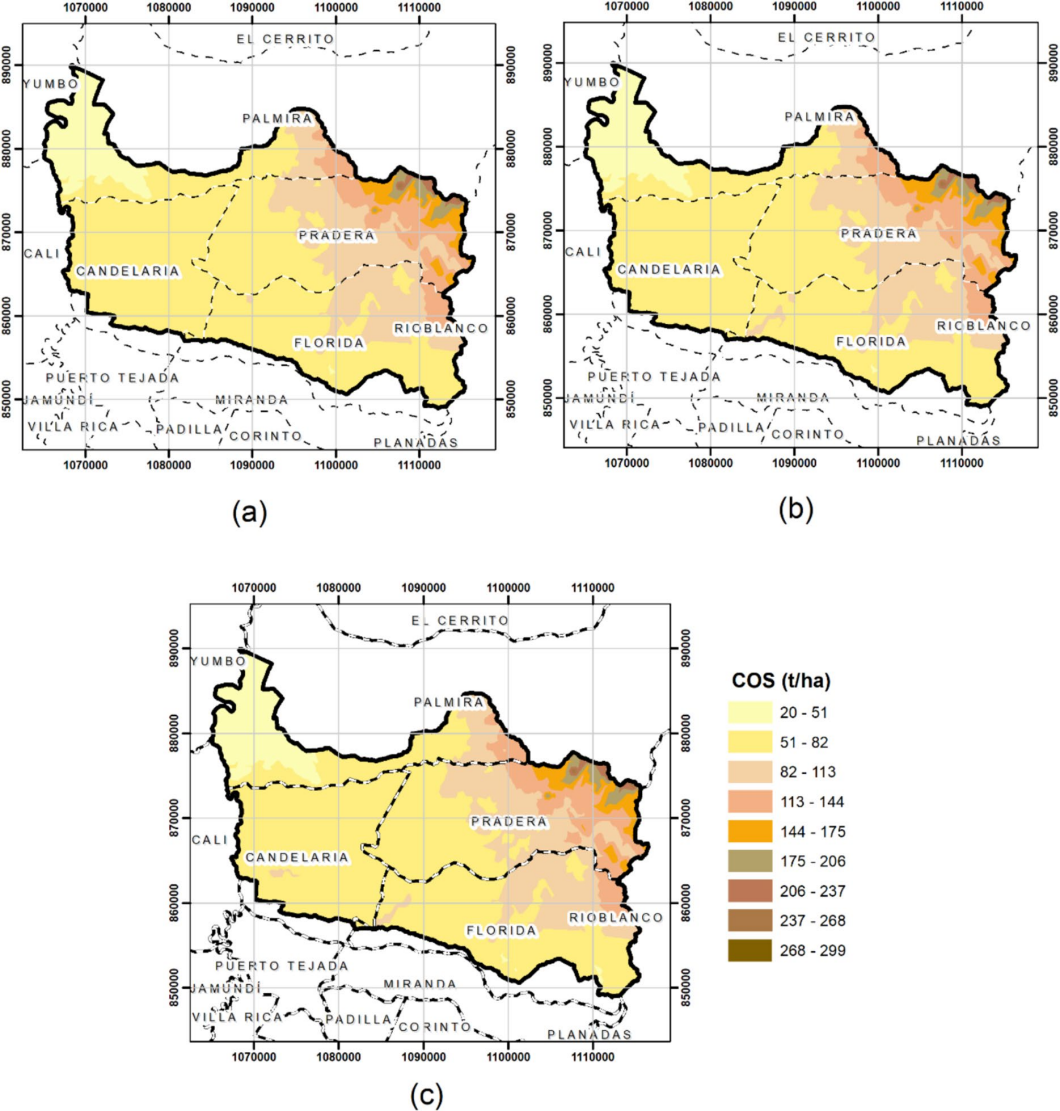
SOC maps for the study area. (**a**) Ordinary univariate kriging. (**b**) Multivariate cokriging (SOC and NT). (**c**) Multivariate cokriging (SOC, pH, and Fe)

The SOC prediction map generated using OK was generated with the values initially calculated at the sampling sites. The SOC map was generated for the CK treatment using total nitrogen (TN), which was the variable with the highest correlation. Finally, the SOC map was generated using CK using the variables that had moderate correlations, which were pH and Fe (Fig. 4). The variables used for CK were adjusted to a normal distribution through logarithmic transformation of the data.

**Fig. 5:**
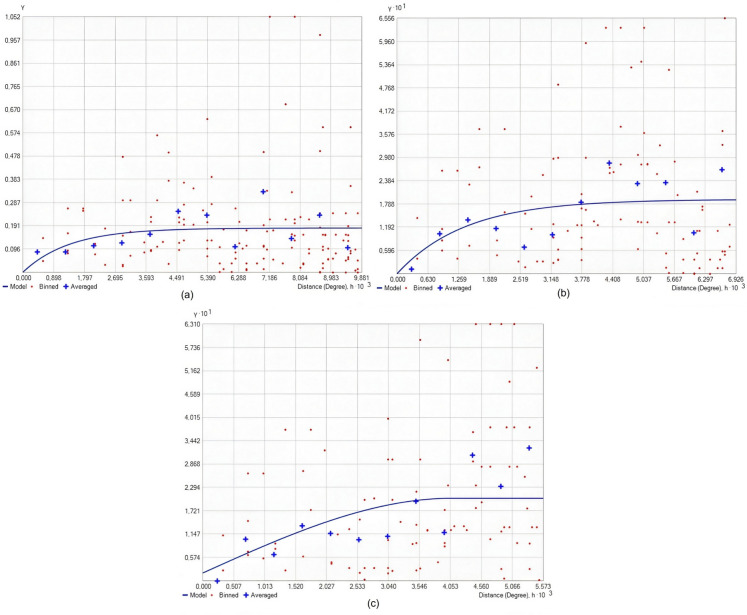
Semivariograms of SOC. (**a**) OK (SOC), (**b**) CK (SOC and NT), and (**c**) CK (SOC, pH, and Fe)

The semivariograms for the OK method exhibit low semivariance at short distances, indicating high similarity between close points. As distance increased, semivariance increased, suggesting greater differences in SOC values between more distant points (Fig. 5). This behavior confirms that there is spatial autocorrelation within the dataset, with points closer together having more similar SOC values.

Compared with OK semivariograms, CK semivariograms exhibit greater mapping accuracy. Both methods have a low semivariance at short distances, and semivariance increases with distance. The exponential model fitted to these semivariograms enhances the prediction accuracy by incorporating additional variables. In the CK + NT treatment, the nugget effect was slightly greater than in the control, which may indicate greater variability in the NT data or better integration of the spatial variability of the SOC. The CK treatment combined with pH and Fe had a smaller nugget effect, indicating reduced unexplained variability and better integration of auxiliary variables. In general, the inclusion of auxiliary variables mitigates the nugget effect and provides a more precise instrument for predicting SOC in soils. This finding demonstrates the robustness and accuracy of the CK versus OK technique (Mallik et al., 2022; Zhu et al., 2018).

In the Guachal watershed, the OK and CK models result in SOC contents ranging from 20 to 299 t ha^−1^. The amount and distribution of SOC were evaluated considering slope, soil order, microclimate, and soil cover. The highest SOC levels were found in the northeastern part of the Guachal watershed. Similarly, a gradient of decrease in SOC is observed from the east to the west. This may be due to edaphic factors, such as soil order and soil cover, in each zone.

**Fig. 6:**
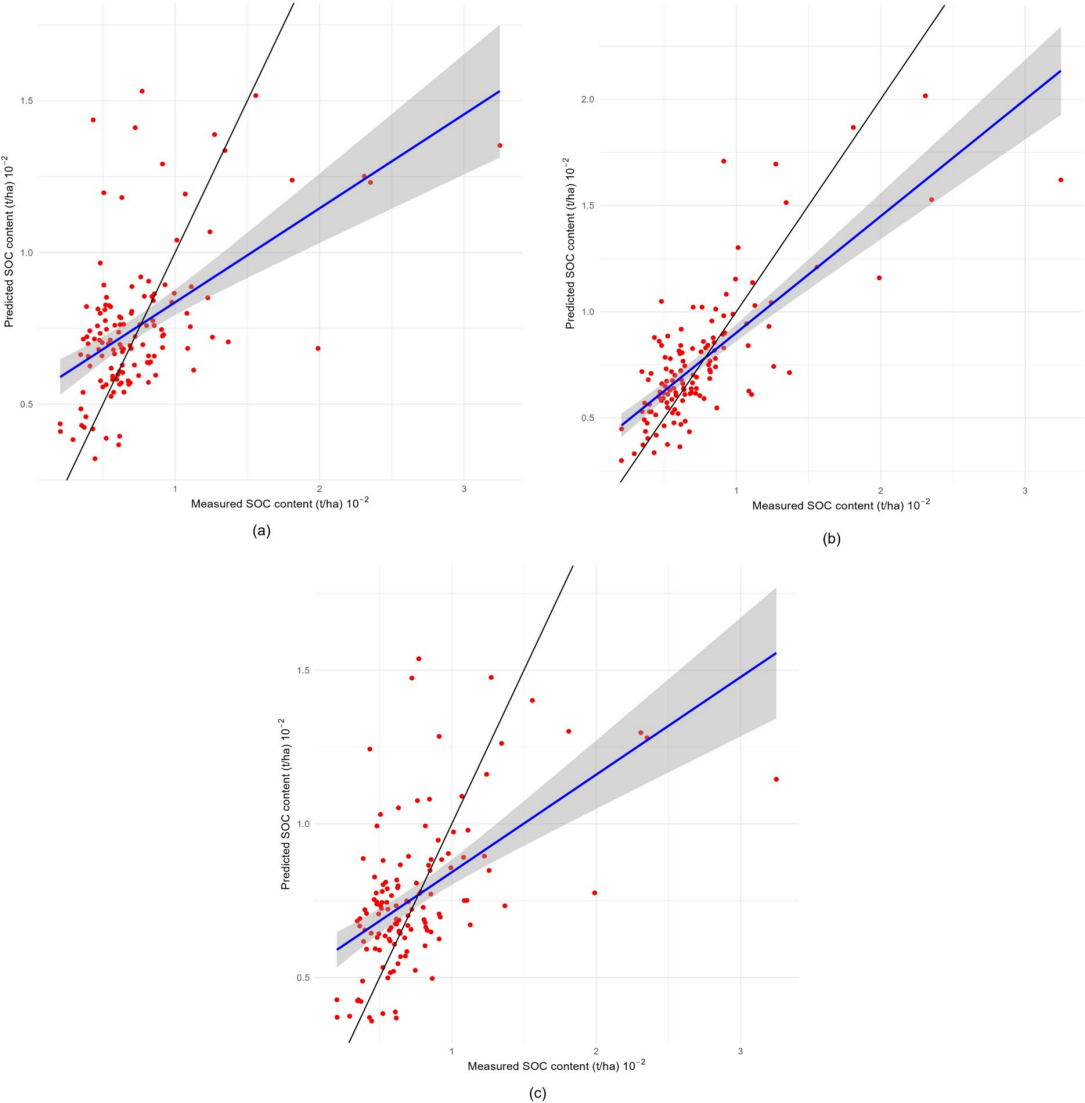
Comparison of predicted and observed SOC content (t ha^−^.^1^) with the perfect fit line (gray) and the best fit line (blue). (**a**) OK (SOC), (**b**) CK (SOC and NT), and (**c**) CK (SOC, pH, and Fe)

Figure 6 shows the scatterplot of the measured SOC content versus the predicted SOC content in the models. Cross-validation of OK using only SOC data indicates that the predicted values tend to underestimate the measured values. The regression line (blue) is significantly different from the 1:1 line (gray), indicating a lack of precision in the predictions. The scatterplot of the points around the regression line suggests considerable variability not captured by the model, reflecting limitations in the ability of the OK to predict SOC values accurately.

The cross-validation plot for CK, which included SOC and total nitrogen (TN), showed enhanced prediction accuracy. The regression line (blue) is closer to the 1:1 line (gray), indicating that the inclusion of TN as an auxiliary variable improves the accuracy of the model. However, the scatter of the points in the CK data suggested that there was variability in the data that the model could not capture.

Cross-validation of CK using SOC with pH and Fe showed greater prediction accuracy than did the other two methods. The regression line (blue) is more aligned with the 1:1 line (gray), indicating better agreement between the measured and predicted values. The lower scatter around the regression line suggested that the inclusion of pH and Fe as auxiliary variables improved the spatial variability of SOC, resulting in more accurate predictions. The cross-validation comparison demonstrated that the use of CK with multiple auxiliary variables provided greater accuracy in terms of soil organic carbon predictions, whereas OK had significant limitations. The incorporation of auxiliary variables via CK substantially improved the accuracy and reduced the variability of the predictions, demonstrating the effectiveness of multivariate techniques in SOC geostatistical mapping.

The analysis of the SOC classification revealed significant variation in the observed contents. According to the classification of Loayza et al. (2020), the highest proportion of SOC was found in the high category, with 47.69% of the sites. The second most common category is very high, at 26.92%. The medium category represented 23.08% of the observations, while the low category accounted for 2.31%. These results indicate that most of the evaluated soils present very high and high SOC stocks, which may suggest good soil management and sustainable agricultural practices in the study area.

The spatial distribution of SOC can be altered by the topography, climate, and hydrology of the region. For example, terrain with lower slopes and good water retention tends to encourage organic matter accumulation and higher SOC levels (Francaviglia et al., 2023). Therefore, areas with higher concentrations may benefit from such conditions. Areas with lower SOC values may indicate more eroded soils, soils overexploited by intensive agricultural practices, or natural less fertile soils. Sustainable soil management in these areas is essential for improving SOC, which is beneficial not only for agricultural productivity but also for climate change mitigation (Rumpel & Chabbi, 2021).

The OK and CK analyses for predicting SOC revealed some variability in the observed and predicted data (Table 3). The OK indicates a smaller standard deviation in the predictions than in the measured values, indicating a generalized underestimate of the spatial variability in SOC. However, the maximum predicted value and maximum error suggest that the OK can produce reasonable estimates in areas with high SOC values, although there is a considerable margin of error.

**Table 3:** Descriptive statistics of measured and predicted SOC content with a logarithmic transformation up to 30 cm of soil in the study area

		**Measured**	**Predicted**	**Error**	**StdError**	**Stdd_Error**	**NormValue**
Ordinary kriging (OK)—SOC	Standard deviation	41.79	24.32	35.44	13.01	0.83	9.99E − 01
	Media	74.25	75.60	1.35	35.85	0.02	0
	Kurtosis	12.26	1.78	8.22	1.70	3.84	− 9.46E − 02
	Skewness	2.94	1.26	− 1.80	1.17	− 1.42	− 3.32E − 17
	Max	324.80	153.13	100.40	80.32	1.44	2.665285
	Min	20.42	32.03	− 189.54	12.51	− 3.91	− 2.665285
Cokriging (CK)—SOC and TN	Standard deviation	41.79	30.08	27.11	13.51	0.73	9.99E − 01
	Media	74.25	75.96	1.70	31.52	0.06	0
	Kurtosis	12.26	4.23	11.16	3.90	2.04	− 9.46E − 02
	Skewness	2.94	1.79	− 2.13	1.74	− 1.14	6.99E − 18
	Max	324.80	201.58	79.58	85.51	1.57	2.665285
	Min	20.42	29.89	− 162.79	11.30	− 2.57	− 2.665285
Cokriging (CK)—SOC, pH, and Fe	Standard deviation	41.79	24.05	34.87	13.51	0.79	9.99E − 01
	Media	74.25	76.08	1.83	37.63	0.04	0
	Kurtosis	12.26	1.41	11.71	1.23	4.02	− 9.46E − 02
	Skewness	2.94	1.06	− 2.32	0.99	− 1.48	− 1.75E − 17
	Max	324.80	153.74	81.07	78.49	1.50	2.665285
	Min	20.42	35.81	− 210.28	13.62	− 3.50	− 2.665285

*Measured*; measured SOC content, *Predicted*; predicted SOC content, *Error*; difference between measured and predicted values, *StdError*; standard error, *Stdd*;_Error standardized standard error, *NormValue*; normalized value

CK with SOC and NT resulted in a significant improvement in standard error reduction compared to OK. This reduction is attributed to the incorporation of total nitrogen, which provides additional information that refines the estimates. The kurtosis and skewness of the predictions indicate more accurate and less skewed distributions, respectively, suggesting that CK can better capture local variations in soil properties. However, the minimum error indicates that there are still significant negative predictions that must be evaluated to avoid misinterpretation.

CK, which included SOC, pH, and Fe, exhibited even greater accuracy in terms of several metrics. However, the standard deviation of the error was slightly greater in the CK treatment than in the other treatments, which included only SOC and NT. The inclusion of pH and Fe provides a more robust framework for estimation purposes. The kurtosis and skewness indicate a less skewed and more concentrated error distribution, respectively. Table A2 contains the results and analysis database (Supplementary Material).

Table 4 provides cross-validation results for residual interpolation models using the ordinary kriging (OK) and cokriging (CK) methods. Different auxiliary variables, such as SOC, TN, pH, and Fe, were incorporated into the analysis. The analyses included the mean (ME), root-mean-square error (RMSE), mean standardized error (MSE), root mean standardized square error (RMSS), and average standard error (ASE). The ME values indicate that all the models slightly overestimate the SOC values, with CK (SOC, pH, and Fe) having the highest bias. In terms of RMSE, CK (SOC and TN) provided the most accurate predictions with the lowest absolute error. The MSE results show minimal differences between the models, with OK having the lowest value.

**Table 4:** Cross-validation measurements of residual interpolation for the models

Measure	OK	CK (SOC and NT)	CK (SOC, pH, and Fe)
Samples	130	130	130
ME	1.34	1.70	1.83
RMSE	35.33	27.06	34.78
MSE	0.02	0.05	0.03
RMSS	0.82	0.73	0.78
ASE	38.12	34.27	39.96

*ME*; mean, *RMSE*; root mean square, *MSE*; mean standardized, *RMSS*; root-mean-square standardized, *ASE*;average standard error

The RMSS and ASE validate the relative accuracy and consistency of the models. The CK model (SOC and TN) had the lowest RMSS and ASE values, indicating better relative accuracy and consistent predictions compared to those of the other models. Although CK (SOC, pH, and Fe) had slightly greater ASE and ME values than did the other treatments, it is a valid alternative due to its ability to capture additional variability. Although the OK method is useful, it has significant limitations compared to multivariate CK models, as evidenced by its higher RMSE and RMSS. The results emphasize the importance of incorporating auxiliary variables to improve the precision and accuracy of geostatistical mapping SOC predictions.

### Limitations and uncertainties of mapping

**Fig. 7:**
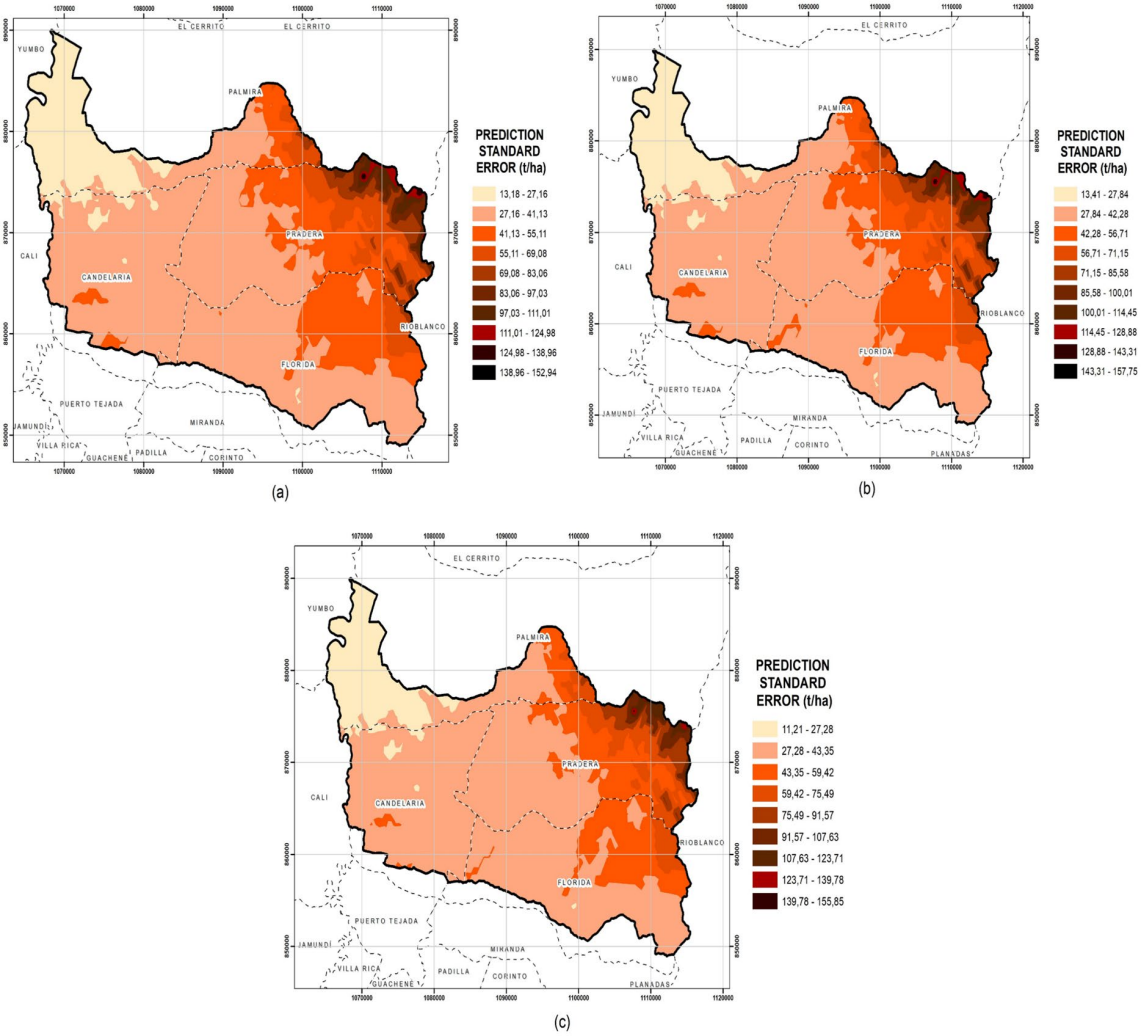
Maps of the prediction standard error of the SOC (t ha.^−1^). (**a**) kriging (OK), (**b**) cokriging (CK) (SOC and NT), and (**c**) cokriging (CK) (SOC, pH, and Fe)

The standard error prediction map for the Guachal watershed is presented in Fig. 7. A standard error surface map illustrates the typical dispersion of estimates at each point on the map. This dispersion is represented by the standard error, which essentially measures the spread of estimates around the average value at each specific site. A high standard error indicates that the prediction made for that particular point has less certainty. Standard error is often used to establish ranges within which the true value for the estimated location is likely to lie (ArcGIS Pro, 2023). The standard error may increase due to the inherent variability of SOC in the landscape or due to the uncertainty associated with the prediction model used. The darker areas indicate that the standard deviation in the SOC prediction is greater. The above results could be due to factors such as soil heterogeneity or greater variability in soil management practices (Minasny & McBratney, 2016).

The standard error maps of SOC prediction in the Guachal watershed revealed significant variations in the precision of the estimates when diverse variables were employed. The standard error values for OK were greater than those obtained for CK, with values ranging from 13.18 to 152.94 t ha^−1^. These findings indicate that the utilization of a single variable (OK) is insufficient for adequately capturing the spatial variability of SOC, resulting in a decrease in precision.

The standard error maps for SOC and NT indicate a significant improvement in prediction accuracy, with standard errors ranging from 13.41 to 157.75 t ha^−1^. Although the maximum error values are high, the inclusion of total nitrogen as a variable contributes to reducing the errors in most of the study area, improving the reliability of the SOC estimates. The standard error maps for SOC, pH, and Fe are shown in Fig. 7c. This map shows a more accurate and precise standard error distribution, with values ranging from 11.21 to 155.85 t ha^−1^. The incorporation of pH and Fe as additional variables improved the capture of soil spatial complexities, reducing errors in SOC predictions. The multivariate approach proves to be the most efficient in terms of accuracy, highlighting the significance of incorporating multiple factors in the geostatistical analysis of SOC.

This study provides valuable information about the drivers of SOC variability in the Guachal watershed and confirms the initial hypothesis. The first hypothesis allowed us to establish a significant relationship between the SOC content in the soil and the order, uses, and environmental conditions. The Andisols had the highest SOC stocks (174.83 t ha^−1^), characterized by their volcanic origin and high organic matter retention capacity, especially in areas with steep slopes and cold climates. Mollisol soils located in flat areas with intensive agricultural practices showed the lowest SOC stocks (20.42 t ha^−1^), which demonstrated the detrimental effects of conventional plowing and monocultures on SOC retention. The second hypothesis was validated by finding that the use of geostatistical methods with auxiliary variables improves SOC prediction accuracy. Cokriging, which includes total nitrogen, pH, and Fe as auxiliary variables, outperformed ordinary kriging in capturing spatial variability and reducing prediction errors. These findings emphasize the importance of incorporating soil properties and environmental variables into geostatistical models to improve prediction accuracy and inform sustainable management practices.

## Conclusions

The results of this study confirm that the variability in SOC contents was strongly influenced by soil properties, land use, and climate. SOC mapping of agricultural soils in the Guachal watershed in Valle del Cauca, Colombia, revealed a high spatial variability in SOC values, ranging from 20 to 294 t ha^−1^. Andisols were the soils with the highest SOC contents due to their volcanic origin. The lowest SOC values were associated with Vertisols and Alfisols, likely due to their dense structure and low permeability. Fine soil textures, such as clay loam, generally result in higher SOC content, indicating a higher capacity for organic matter retention. Steep slope soils showed high SOC stocks, probably due to the accumulation of organic matter in natural depressions. Soils in the flat zone with intensive agricultural practices presented the lowest SOC values. In terms of soil cover, fragmented mixed forests and cultivated pastures had the highest SOC values, indicating the importance of vegetation cover for carbon capture and storage. The incorporation of auxiliary variables into the geostatistical analysis improves prediction accuracy in SOC mapping. The cokriging method, which included total nitrogen, pH, and Fe in the analysis, demonstrated higher accuracy than ordinary kriging.

This research enabled us to establish the SOC baseline and its relationship with different environmental variables. The analysis of soil carbon content in agriculture allows for the adoption of soil management strategies to optimize carbon sequestration and improve environmental sustainability in the region. Furthermore, it is necessary to establish soil conservation policies that consider the spatial heterogeneity of soils to maximize ecological and agricultural benefits.

## Supplementary information

Below is the link to the electronic supplementary material.

ESM 1 (PDF 95.9 KB)

ESM 2 (PDF 90.9 KB)

## Data Availability

No datasets were generated or analysed during the current study.
