# Supplementary material for: Soil organic carbon in agricultural soils of an inter-Andean valley in Colombia: understanding the effects of environmental and geographic variables
Source: Environ Monit Assess. 2025 May 30;197(6):697. doi: 10.1007/s10661-025-14123-1 (PMC12125077; doi:10.1007/s10661-025-14123-1)
Supplement: Supplementary file 1 — (PDF 95.9 KB) [file 10661_2025_14123_MOESM1_ESM.pdf]

Table A1. Database of soil physicochemical properties and environmental variables - Guachal watershed, Colombia.

ID	Longitude	Latitude	Soil Order	Microclimate	Slope	Soil Cover	Height	Sand	Clay
1	-76,193823	3,284099574	Entisoles	Temperate or Medium (24-18 °C)	ST	CA	1266	35,87	34,85
2	-76,114785	3,437090996	Andisoles	Cool (18-12 °C)	ST	STV	2677	34,43	24,54
3	-76,269572	3,346425856	Mollisols	Hot (>24 °C)	Fl	SC	1014	57,82	15,64
4	-76,235389	3,307909096	Mollisols	Hot (>24 °C)	Sl	SC	1054	54,03	13,51
5	-76,345337	3,315122587	Mollisols	Hot (>24 °C)	Fl	SC	984	37,91	35,27
6	-76,294136	3,350833768	Mollisols	Hot (>24 °C)	Fl	SC	999	53,98	17,59
7	-76,353114	3,331583903	Mollisols	Hot (>24 °C)	Fl	SC	981	67,59	18,31
8	-76,308139	3,362315319	Mollisols	Hot (>24 °C)	Fl	SC	995	51,8	19,68
9	-76,39489	3,40401452	Mollisols	Hot (>24 °C)	Fl	SC	960	25,08	23,83
10	-76,316599	3,371425052	Mollisols	Hot (>24 °C)	Fl	SC	992	53,11	20,49
11	-76,379498	3,395547992	Mollisols	Hot (>24 °C)	Fl	COINV	965	37,07	25,99
12	-76,241214	3,48431437	Mollisols	Hot (>24 °C)	Sl	SC	1043	48,37	24,41
13	-76,095403	3,440942977	Andisoles	Cool (18-12 °C)	ST	STV	2690	57,8	12,32
14	-76,257356	3,507098322	Vertisols	Hot (>24 °C)	Sl	SC	1017	11,84	53,47
15	-76,24384	3,449252633	Mollisols	Hot (>24 °C)	Sl	SC	1050	16,76	52,3
16	-76,405767	3,407630368	Mollisols	Hot (>24 °C)	Fl	SC	958	60,11	11,48
17	-76,227757	3,295475555	Mollisols	Hot (>24 °C)	Sl	SC	1107	60,2	17,53
18	-76,345882	3,431811468	Mollisols	Hot (>24 °C)	Fl	SC	969	56,38	29,14
19	-76,4502	3,409087578	Vertisols	Hot (>24 °C)	Fl	SC	948	29,67	36,9
20	-76,443221	3,500082269	Mollisols	Hot (>24 °C)	Fl	SC	945	51,48	44,32
21	-76,330693	3,476780406	Vertisols	Hot (>24 °C)	Fl	SC	971	54,21	35,43
22	-76,420897	3,41238426	Mollisols	Hot (>24 °C)	Fl	SC	954	66,2	15,54
23	-76,431986	3,516740758	Vertisols	Hot (>24 °C)	Fl	SC	947	71,2	22,67
24	-76,097398	3,432788034	Andisoles	Cool (18-12 °C)	ST	STV	2773	61,77	9,08
25	-76,352976	3,35550344	Mollisols	Hot (>24 °C)	Fl	SC	980	53,35	30,48
26	-76,450775	3,559342493	Vertisols	Hot (>24 °C)	Fl	CP	944	33,93	26,55
27	-76,334392	3,384755912	Mollisols	Hot (>24 °C)	Fl	SC	983	64,69	18,78
28	-76,3364	3,456011963	Mollisols	Hot (>24 °C)	Fl	SC	971	50,45	31,04
29	-76,250859	3,419795992	Mollisols	Hot (>24 °C)	Sl	SC	1041	29,68	21,63
30	-76,344117	3,392623766	Mollisols	Hot (>24 °C)	Fl	SC	978	42,34	24,82
31	-76,362192	3,396659461	Mollisols	Hot (>24 °C)	Fl	SC	969	64,25	11,46

ID	Silt	TEXTURE	DA	pH	CE	MO	CO	Total Nitrogen	S	B	P	Ca
1	29,28	CL	1,09	6,42	0,2	4,81	2,79	0,32	10,76	0,48	7,54	33,41
2	41,03	L	0,46	5,96	0,45	5,36	3,11	0,25	10,44	0,15	87,15	5,76
3	26,54	SL	1,76	5,9	0,16	4,12	2,39	0,16	4,73	0,24	15,85	7,03
4	32,46	SL	1,63	6,33	0,14	2,88	1,67	0,18	3,37	0,17	14,2	4,91
5	26,82	CL	1,40	7,21	0,16	1,93	1,12	0,13	4,53	0,24	31,24	18,58
6	28,43	SL	1,59	7,49	0,18	2,33	1,35	0,12	2,7	0,12	117,09	9,37
7	14,1	SL	1,47	6,3	0,17	2,64	1,53	0,1	6,95	0,26	105,22	5,22
8	28,52	L	1,58	6,97	0,16	1,26	0,73	0,15	3,55	0,31	10,31	7,84
9	51,09	SiL	1,42	7,25	0,24	2,29	1,33	0,17	4,4	0,29	111,98	12,58
10	26,4	SCL	1,46	7,59	0,22	2,26	1,31	0,1	8,75	0,14	25,64	10,69
11	36,94	L	1,35	7,58	0,31	2,57	1,49	0,21	5,93	0,44	73,28	14,32
12	27,22	SCL	1,32	7,1	0,22	2,41	1,4	0,17	7,24	0,57	77,58	21,21
13	29,88	SL	0,46	6,31	0,89	11,31	6,56	0,7	37,01	0,62	41,79	19,15
14	34,69	C	1,21	7,18	0,19	3,31	1,92	0,22	5,88	0,26	6,99	27,52
15	30,94	C	1,12	7,28	0,18	4,05	2,35	0,22	4,53	0,47	10,17	35,86
16	28,41	SL	1,60	7,98	0,22	2,14	1,24	0,13	3,7	0,31	59,63	8,85
17	22,27	SL	1,73	7,18	0,11	1,36	0,79	0,12	3,52	0,19	3,77	3,38
18	14,48	SCL	1,60	8,12	0,38	2,69	1,56	0,14	9,72	0,21	31,11	20,84
19	33,43	CL	1,46	7,4	0,2	1,53	0,89	0,18	5,17	0,27	4,56	11,38
20	4,2	SC	1,35	7,52	0,64	2,62	1,52	0,19	18,74	0,54	21,7	22,98
21	10,36	SC	1,44	8,22	0,34	2,22	1,29	0,14	4,71	0,19	48,02	30,51
22	18,26	SL	1,64	6,77	0,19	2,26	1,31	0,13	4,03	0,24	56,02	7,8
23	6,13	SCL	1,63	8,81	0,72	1,52	0,88	0,1	20,81	0,47	51,05	22,29
24	29,15	SL	0,97	5,5	0,13	4,29	2,49	0,2	8,54	0,48	18,11	5,39
25	16,17	SCL	1,61	6,92	0,16	2,57	1,49	0,13	5,86	0,16	30,17	9,5
26	39,52	L	1,55	8,6	0,91	1,09	0,63	0,11	27,66	0,2	61,94	19,83
27	16,53	SL	1,53	7,72	0,15	2,36	1,37	0,13	2,75	0,17	19,81	17,44
28	18,51	SCL	1,37	7,74	0,22	2,07	1,2	0,12	6,12	0,21	28,83	17,49
29	48,69	L	1,46	5,8	0,72	2,47	1,43	0,15	8,86	0,26	17,66	8,49
30	32,84	L	1,32	7,62	0,22	2,76	1,6	0,13	5,79	0,28	88,35	16,16
31	24,29	SL	1,53	6,14	0,44	1,79	1,04	0,13	3,86	0,33	9,85	5,71

ID	Mg	K	Na	Fe	Cu	Mn	Zn	CICA	COS
1	13,51	0,14	0,37	33,42	3,51	4,76	0	44,06	91,23
2	1,61	0,3	0	46,04	1,33	2,81	3,07	29,23	43,27
3	1,98	0,35	0	299,96	1,67	6,46	3,06	15,47	125,86
4	1,4	0,24	0	98,09	1,83	15,34	1,91	11,08	81,55
5	7,48	0,31	0,22	53,29	5,91	1,07	0	21,21	46,98
6	2,61	0,13	0	103,33	1,93	2,33	0	13,2	64,28
7	1,53	0,29	0	175,5	1,72	2,13	1,19	8,55	67,43
8	3,08	0,13	0	75,26	2,6	3,04	1,97	14,03	34,56
9	3,33	0,19	0,17	108	3,88	3,06	1,32	15,34	56,66
10	4,82	0,18	0,17	37,99	2,42	1,1	0	13,85	57,27
11	4,7	0,39	0	60,41	4,86	2,91	3,31	18,47	60,31
12	8,47	0,46	0	46,16	6,8	2,57	5,8	32,99	55,32
13	4,19	2,39	0	240,46	5,06	1,81	19,97	29,68	91,27
14	16,58	0,18	0,19	26,5	7,99	1,32	0	45,43	69,59
15	18,28	0,18	0,35	19,57	7,02	1,74	0	56,78	78,69
16	2,69	0,25	0,15	19,65	2,83	1,87	0	11,67	59,65
17	0,9	0	0	82,79	0	13,49	0	6,45	41,01
18	12,3	0,31	0,49	13,34	5,86	0	0	22,64	74,72
19	9,56	0,14	0	21,33	3,36	2,45	0	24,16	38,93
20	16,86	0,29	0,58	20,34	8,99	1,97	0	31,31	61,56
21	6,62	0,26	0,54	18,83	5,25	0	0	25,14	55,70
22	2,93	0,31	0	33,96	1,94	3,85	0	8,93	64,55
23	18,42	1,09	0,7	11,25	5,64	9,12	0	12,87	43,03
24	2,99	0,18	0	413,66	3,05	1,85	2,63	22,76	72,37
25	2,52	0,16	0	92,77	2,9	1,68	0	12,81	71,79
26	22,72	0,87	0,91	19,97	3,03	2,8	0	15,31	29,30
27	7,99	0,21	0,28	26,81	4,9	0	0	22,14	62,75
28	4,72	0,19	0,3	39,98	9,19	0	0	17,91	49,35
29	2,81	0,14	0	104,67	11,34	7,48	1,29	13,39	62,84
30	7,22	0,53	0	32,93	4,23	1,31	0	18,18	63,46
31	1,84	0,13	0	51,15	1,65	6,45	0	8,81	47,66

ID	Longitude	Latitude	Soil Order	Microclimate	Slope	Soil Cover	Height	Sand	Clay
32	-76,356656	3,37434937	Mollisols	Hot (>24 °C)	Fl	SC	977	55,99	23,14
33	-76,351657	3,418431857	Mollisols	Hot (>24 °C)	Fl	SC	971	46,79	18,54
34	-76,209252	3,406340264	Mollisols	Hot (>24 °C)	SI	MCP	1167	55,54	13,67
35	-76,184061	3,277951872	Mollisols	Temperate or Medium (24-18 °C)	Fl	FMFNV	1349	41,85	25,68
36	-76,243611	3,38314457	Entisoles	Hot (>24 °C)	Fl	PN	1045	28,87	41,55
37	-76,268515	3,356006284	Mollisols	Hot (>24 °C)	Fl	SC	1517	28,33	31,13
38	-76,193555	3,308591114	Entisoles	Temperate or Medium (24-18 °C)	ST	CPW	1426	18,64	55,91
39	-76,197616	3,30903746	Entisoles	Temperate or Medium (24-18 °C)	ST	CPW	1357	33,16	43,39
40	-76,191374	3,307761722	Entisoles	Temperate or Medium (24-18 °C)	ST	STV	1468	48,15	24,18
41	-76,18364	3,277611121	Mollisols	Temperate or Medium (24-18 °C)	Fl	CP	1351	51,12	15,83
42	-76,216463	3,31268698	Mollisols	Hot (>24 °C)	SI	STV	1090	50,69	20,97
43	-76,120971	3,441110963	Andisoles	Cool (18-12 °C)	ST	STV	2572	43,04	14,29
44	-76,145314	3,443729339	Andisoles	Cool (18-12 °C)	SB	FMFCP	1846	25,37	45,89
45	-76,188277	3,31030377	Entisoles	Temperate or Medium (24-18 °C)	ST	MCP	1571	26,77	49,79
46	-76,20336	3,309046341	Entisoles	Temperate or Medium (24-18 °C)	ST	STV	1306	37,48	35,75
47	-76,227705	3,299220609	Mollisols	Hot (>24 °C)	SI	SC	1100	56,42	21,45
48	-76,090014	3,431876075	Andisoles	Cool (18-12 °C)	ST	CP	1112	37,54	24,55
49	-76,14523	3,423419922	Andisoles	Cool (18-12 °C)	ST	CPW	1741	55,82	15,22
50	-76,132534	3,446257904	Andisoles	Cool (18-12 °C)	ST	CPW	2253	53,65	11,11
51	-76,219891	3,28366162	Mollisols	Hot (>24 °C)	Inc	SC	1112	42,56	22,97
52	-76,121413	3,45517385	Andisoles	Cool (18-12 °C)	ST	CP	2466	41,33	10,5
53	-76,120016	3,43591497	Andisoles	Cool (18-12 °C)	ST	CP	2582	34,3	11,99
54	-76,219448	3,283110914	Mollisols	Hot (>24 °C)	Inc	SC	1114	39,83	23,66
55	-76,195517	3,28442216	Mollisols	Temperate or Medium (24-18 °C)	Fl	CP	1255	49,6	21,79
56	-76,190824	3,30434186	Entisoles	Temperate or Medium (24-18 °C)	ST	STV	1441	12,92	28,82
57	-76,205119	3,390344376	Entisoles	Hot (>24 °C)	Fl	FMFNV	1140	66	9,65
58	-76,205327	3,49013595	Entisoles	Temperate or Medium (24-18 °C)	ST	FMFNV	1284	41,58	11,88
59	-76,205879	3,307445474	Entisoles	Temperate or Medium (24-18 °C)	ST	STV	1222	61,15	15,57
60	-76,20344	3,28286174	Entisoles	Hot (>24 °C)	Fl	FMFCP	1188	49,55	15,9
61	-76,224538	3,533593057	Entisoles	Temperate or Medium (24-18 °C)	SI	STV	1149	55,28	9,13
62	-76,225596	3,281934588	Mollisols	Hot (>24 °C)	Fl	SC	1091	60,61	16,17
63	-76,20128	3,30903677	Entisoles	Temperate or Medium (24-18 °C)	ST	AND	1329	26,5	45,69
64	-76,240859	3,462063362	Entisoles	Hot (>24 °C)	Fl	STV	1054	51,15	11,71

ID	Silt	TEXTURE	DA	pH	CE	MO	CO	Total Nitrogen	S	B	P	Ca
32	20,87	SCL	1,31	7,39	0,42	4,97	2,88	0,29	18,61	0,77	1042,39	22,02
33	34,67	L	1,52	6,23	0,19	2,1	1,22	0,17	4,96	0,26	66,78	10,71
34	30,79	SL	1,16	6,62	0,25	4,15	2,41	0,26	5,59	0,17	27,48	11,3
35	32,47	L	1,35	6,78	0,11	1,5	0,87	0,13	10,46	0,36	3,37	17,39
36	29,58	C	1,13	6,7	0,2	2,95	1,71	0,15	3,5	0,35	63,89	22,37
37	40,54	CL	1,47	6,79	0,16	2,12	1,23	0,14	4,13	0,3	21,45	9,94
38	25,45	C	0,63	6,05	0,18	4,26	2,47	0,25	5,35	0,07	1,38	23,14
39	23,45	C	1,28	6,64	0,14	4,12	2,39	0,3	6,48	0,04	0	23,56
40	27,67	SCL	1,07	6,68	0,17	2,76	1,6	0,2	5,85	0,04	3,18	24,73
41	33,05	L	1,26	6,58	0,13	1,81	0,98	0,16	7,98	0,44	8,9	17,36
42	28,34	L	1,34	6,32	0,15	3,52	2,04	0,2	6,78	0,09	6,31	9,44
43	42,67	L	0,60	5,32	0,11	17,29	10,03	0,77	2,53	0,17	8,51	4,4
44	28,74	C	0,72	7,3	0,54	4,07	2,36	0,22	9,04	0,29	117,39	25,5
45	23,44	C	1,04	6,3	0,18	4,72	2,74	0,23	7,43	0,09	1,59	17,88
46	26,77	CL	1,11	5,51	0,14	4,69	2,72	0,3	11,65	0,07	0	5,43
47	22,13	SCL	1,59	6,21	0,11	2,29	1,33	0,16	7,98	0,43	2,8	5,08
48	37,91	L	0,72	5,85	0,52	10,81	6,27	0,66	11,59	0,23	16,39	15,07
49	28,96	SL	1,03	5,52	0,53	6,93	4,02	0,36	10,98	0,21	59,09	9,74
50	35,24	SL	1,18	5,67	0,36	3,76	2,18	0,24	6,47	0,25	35,73	8,24
51	34,47	L	1,61	6,89	0,19	1,84	1,07	0,15	10,41	0,64	6,54	11,3
52	48,17	L	0,72	5,44	0,87	10,15	5,89	0,68	10,49	0,18	28,57	11,32
53	53,71	SiL	0,86	5,72	0,1	7,17	4,16	0,31	3,07	0,15	13,02	2,4
54	36,51	L	1,43	6	0,22	3,28	1,9	0,24	12,27	0,63	4,7	8,04
55	28,61	L	1,46	6,79	0,11	2,71	1,57	0,16	6,89	0,63	4,15	13,25
56	58,26	SiCL	1,44	6,75	0,16	4,4	2,55	0,18	7,19	0,26	6,48	34,84
57	24,35	SL	1,23	6,92	0,3	4,28	2,48	0,27	6,85	0,46	6,33	38,93
58	46,54	L	0,95	6,15	0,66	6	3,48	0,39	11,5	0,15	35,19	31,8
59	23,28	SL	1,11	6,52	0,13	3,62	2,1	0,2	5	0,14	3,34	8,88
60	34,55	L	1,11	6,06	0,22	5,76	3,34	0,34	4,12	0,15	8,93	27,81
61	35,59	SL	1,23	5,54	0,23	5,03	2,92	0,24	8,74	0,16	2,4	7,48
62	23,22	SL	1,60	6,06	0,15	1,79	1,04	0,13	10,64	0,6	10,58	5,46
63	27,81	C	1,07	6,95	0,21	3,74	2,17	0,26	6,03	0,04	3,23	28,24
64	37,14	L	0,97	6,63	0,59	5,79	3,36	0,32	13,65	0,31	59,48	30,3

ID	Mg	K	Na	Fe	Cu	Mn	Zn	CICA	COS
32	6,7	2,37	0,23	177,66	26,52	1,82	7,89	25,44	112,83
33	4,95	0,23	0	88,52	7,55	10,43	0	16,12	55,76
34	2,19	0,26	0	68,08	2,16	1,09	3,21	17,7	83,95
35	6,52	0,12	0,19	49,92	4,22	2,73	0	25,4	35,33
36	9,72	0,22	0	35,06	6,01	3,8	1,7	37,08	57,99
37	4,41	0,24	0	72,51	4,36	2,29	0	16,98	54,26
38	15,45	0,15	0	31	4,81	18,27	0	48,2	46,55
39	15,84	0,09	0,16	17,8	5,1	6,2	0	45,11	92,03
40	16,76	0,42	0	17,61	3,14	9,27	0	43,92	51,36
41	7,65	0,13	0,21	51,43	3,49	2,74	0	24,99	37,04
42	1,54	0,17	0	107,09	1,85	3,09	0	14,39	82,01
43	1,28	0,21	0	278,42	0	1,67	0	59,21	181,02
44	2,79	0,85	0	118,17	5,08	3,68	2,31	19,17	50,63
45	15,54	0,42	0	33,98	6,84	14,74	0	45,91	85,49
46	5,66	0	0	241,28	4,99	9,94	0	23,98	90,58
47	1,26	0,1	0	92,79	0	5,59	0	9,62	63,44
48	2,35	2,2	0	237,59	2,62	11,49	6,29	33,26	134,51
49	1,73	1,19	0	218,48	2,4	8,37	4,02	18,79	124,12
50	2,5	0,63	0	328,79	1,7	2,38	0	20,33	77,17
51	3,31	0,09	0	43,13	2,3	2,32	2	14,66	51,68
52	1,63	0,98	0	167,42	2,47	9,97	3,49	37,93	127,29
53	0,4	0,2	0	68,64	0	1,22	0	0	107,00
54	2,69	0,21	0	172,52	4,07	5,38	5,86	14,44	81,51
55	8,76	0,22	0	50,36	2,68	2,24	0	21,44	68,70
56	14,84	0,11	0,7	11,59	1,34	1,95	0	50,46	110,52
57	12,42	1,38	0,2	16,77	4,69	2,03	0	48,1	91,26
58	11,54	0,58	0,21	41,94	3,98	9,5	5,31	42,61	99,41
59	6,32	0,09	0	25,17	2,48	8,04	1,29	19,2	70,02
60	11,54	0,25	0,18	48,21	6,35	4,72	4,19	41,13	111,22
61	2,97	0,23	0	120,15	3,73	16,02	1,75	21,6	108,17
62	1,57	0,2	0	154,89	4,35	3,05	1,05	12,38	49,92
63	18,8	0,2	0	12,23	3,14	3,81	0	46,35	69,66
64	15,03	5,08	0,15	69,7	10,56	4,23	20,03	47,32	97,64

ID	Longitude	Latitude	Soil Order	Microclimate	Slope	Soil Cover	Height	Sand	Clay
65	-76,222451	3,50424952	Entisoles	Temperate or Medium (24-18 °C)	ST	FMFNV	1095	49,45	11,12
66	-76,186709	3,27914086	Inceptisols	Temperate or Medium (24-18 °C)	ST	FMFNV	1320	26,66	43,72
67	-76,190428	3,416199094	Entisoles	Temperate or Medium (24-18 °C)	ST	FMFNV	1319	40,55	36,39
68	-76,205061	3,283164101	Entisoles	Hot (>24 °C)	Fl	CPA	1194	41,05	30,15
69	-76,180104	3,42174925	Entisoles	Temperate or Medium (24-18 °C)	ST	CP	1351	51,89	13,1
70	-76,219766	3,489002901	Entisoles	Temperate or Medium (24-18 °C)	SI	CP	1196	21,65	43,87
71	-76,191984	3,283255948	Entisoles	Temperate or Medium (24-18 °C)	ST	CA	1289	58,03	19,29
72	-76,242206	3,324428797	Entisoles	Hot (>24 °C)	Fl	CP	1028	12,1	31,87
73	-76,19635	3,400313872	Inceptisols	Temperate or Medium (24-18 °C)	Inc	STV	1472	45,12	19,49
74	-76,187662	3,477076245	Inceptisols	Temperate or Medium (24-18 °C)	ST	STV	1734	33,93	24,72
75	-76,194688	3,28456461	Mollisols	Temperate or Medium (24-18 °C)	Fl	CA	1256	40,98	32,25
76	-76,19827	3,28403406	Mollisols	Temperate or Medium (24-18 °C)	Fl	CP	1240	43,79	30,91
77	-76,211862	3,533611921	Inceptisols	Temperate or Medium (24-18 °C)	SB	FMFCP	1300	32,52	41,9
78	-76,204274	3,47351999	Inceptisols	Temperate or Medium (24-18 °C)	Inc	CP	1295	21,11	29,4
79	-76,452625	3,499775601	Inceptisols	Hot (>24 °C)	Fl	SC	945	17,33	41,69
80	-76,464759	3,448614022	Inceptisols	Hot (>24 °C)	Fl	CP	946	61,74	30,06
81	-76,229157	3,500283689	Inceptisols	Temperate or Medium (24-18 °C)	SI	CP	1084	27,3	51,1
82	-76,473885	3,492608127	Inceptisols	Hot (>24 °C)	Fl	SC	945	54,66	45,34
83	-76,181483	3,321295423	Inceptisols	Temperate or Medium (24-18 °C)	ST	CPW	1716	26,49	41,7
84	-76,19651	3,40963687	Inceptisols	Temperate or Medium (24-18 °C)	Inc	CP	1248	28,92	38,43
85	-76,171048	3,41882031	Inceptisols	Temperate or Medium (24-18 °C)	ST	CP	1459	66,06	9,49
86	-76,458084	3,434041421	Inceptisols	Hot (>24 °C)	Fl	SC	947	22,37	30,22
87	-76,081103	3,433895898	Andisoles	Cool (18-12 °C)	ST	STV	2820	19,43	39,55
88	-76,460029	3,560885811	Inceptisols	Hot (>24 °C)	Fl	SC	944	16,81	47,91
89	-76,457881	3,42315476	Inceptisols	Hot (>24 °C)	Fl	CP	952	27,36	31,9
90	-76,210721	3,30718825	Mollisols	Hot (>24 °C)	SI	STV	1150	51,08	26,83
91	-76,196666	3,28443033	Mollisols	Temperate or Medium (24-18 °C)	Fl	FMFCP	1243	65,46	15,88
92	-76,211777	3,283226721	Mollisols	Hot (>24 °C)	Inc	SC	1141	56,62	16,98
93	-76,421747	3,552468075	Mollisols	Hot (>24 °C)	Fl	CP	948	58,68	33,09
94	-76,423695	3,486012994	Vertisols	Hot (>24 °C)	Fl	SC	948	57,13	16,45
95	-76,198922	3,28430862	Mollisols	Temperate or Medium (24-18 °C)	Fl	CA	1225	26,26	42,79
96	-76,239957	3,46943924	Mollisols	Hot (>24 °C)	SI	CPA	1050	32,05	43,57
97	-76,339541	3,304830794	Mollisols	Hot (>24 °C)	Fl	SC	987	56,76	32,99

ID	Silt	TEXTURE	DA	pH	CE	MO	CO	Total Nitrogen	S	B	P	Ca
65	39,43	L	1,48	5,5	0,12	1,5	0,87	0,09	4,13	0,14	22,84	8,73
66	29,62	C	1,01	6,22	0,12	2,74	1,59	0,23	9,2	0,54	0	18,71
67	23,06	CL	1,29	7,31	0,43	2,26	1,31	0,18	10,64	0,21	5,71	42,08
68	28,8	CL	1,27	6,47	0,17	5,55	3,22	0,29	11,82	0,8	2,17	15,47
69	35,01	L	1,40	6,53	0,22	3,36	1,95	0,23	5,34	0,23	7,87	13,71
70	34,48	C	0,94	5,38	0,13	4,65	2,7	0,36	12,35	0,44	2,25	4,47
71	22,68	SL	1,18	6,96	0,15	2	1,16	0,19	5,47	0,24	4,81	30,82
72	56,03	SiCL	1,24	7,02	0,23	2,22	1,29	0,23	7,16	0,26	21,02	13,48
73	35,39	L	1,23	6,96	0,35	3,74	2,17	0,29	6,45	0,4	4,98	18,56
74	41,35	L	1,46	6,68	0,12	2,48	1,44	0,19	6,71	0,44	1,55	23,15
75	26,77	CL	1,28	6,67	0,12	4,88	2,83	0,18	9,18	0,3	1,36	13,49
76	25,3	CL	1,07	6,67	0,22	1,97	1,14	0,15	8,76	0,6	10,06	33,25
77	25,58	C	0,90	5,87	0,22	5,95	3,45	0,32	5,09	0,05	3,42	15,23
78	49,49	CL	1,42	5,9	0,26	8,05	4,67	0,54	5,49	0,1	2,42	17,22
79	40,98	SiC	1,30	7,33	0,2	2,31	1,34	0,16	6,01	0,29	33,68	17,43
80	8,2	SCL	1,37	6,65	0,24	2,19	1,27	0,21	14,86	0,32	15,5	9,76
81	21,6	C	1,07	6,45	0,12	3,88	2,25	0,2	3,39	0,03	1,97	14,01
82	0	SC	1,15	4,94	0,88	1,81	1,05	0,18	13,34	0,21	5,28	9,02
83	31,81	C	1,06	5,23	0,13	4,6	2,67	0,31	11,93	0,03	2,08	7,84
84	32,65	CL	1,06	6,45	0,15	4,64	2,69	0,31	2,88	0,1	27,62	31,14
85	24,45	SL	0,91	6,67	0,27	3,03	1,76	0,22	4,88	0,41	43,82	8,88
86	47,41	CL	1,37	5,64	0,35	2,59	1,5	0,28	20,2	0,27	15,27	6,83
87	41,02	SiCL	1,07	5,57	0,49	8,4	4,87	0,6	6,84	0,23	38,47	5,48
88	35,28	C	1,44	7,5	0,22	1,78	1,03	0,16	6,34	0,31	7,72	13,65
89	40,74	CL	1,24	6,53	0,41	2,84	1,65	0,26	16,72	0,48	95,3	8,91
90	22,09	SCL	1,37	6,18	0,16	2,62	1,52	0,21	6,58	0,06	3,3	10,65
91	18,66	SL	1,27	7,9	0,52	3,91	2,27	0,16	14,37	0,59	75,91	40,15
92	26,4	SL	1,49	6,77	0,12	2	1,16	0,13	5,94	0,48	4,06	7,96
93	8,23	SCL	1,40	8,5	0,69	2,5	1,45	0,13	8,86	0,17	92,65	36,49
94	26,42	SL	1,38	8,04	1,57	1,45	0,84	0,18	78,87	2,01	20,38	12,86
95	30,95	C	0,97	6,52	0,19	3,4	1,97	0,28	6,6	0,16	2,37	28,35
96	24,38	C	0,95	6,39	0,24	4,24	2,46	0,31	9,77	0,23	3,73	31,28
97	10,25	SCL	1,41	5,59	1,14	2,62	1,52	0,16	27,61	0,24	20,51	11,17

ID	Mg	K	Na	Fe	Cu	Mn	Zn	CICA	COS
65	14,25	0,19	0,27	44,58	3,78	13,82	1,04	20,06	38,65
66	16,35	0,13	0	31,32	6,92	12,94	0	40,33	48,36
67	14,56	0,77	0,31	8,68	1,93	2,19	0	50,07	50,65
68	6,77	0,14	0,14	68,87	7,33	3,57	0	28,75	122,59
69	5,24	0,2	0	101,44	2,7	4,41	2,29	17,26	81,71
70	1,62	0,1	0	98,45	4,68	17,73	0	22,09	76,22
71	12,15	0,25	0,54	10,57	0	3,01	0	51,78	40,93
72	5,15	0,36	0,39	103,45	8,89	2,24	0	19,86	48,11
73	8,08	0,38	0	37,19	3,62	4,27	1,04	25,07	80,22
74	18,99	0,09	0	22,24	3,23	5,19	0	46,67	63,09
75	7,26	0,12	0,17	79,94	8,32	2,8	0	20,94	108,67
76	14,52	0,7	0,36	24,84	4,05	3,32	1,99	44,24	36,47
77	7,7	0,14	0	107,05	8,79	5,16	2,95	33,6	93,02
78	6,67	0,16	0	95,09	7,55	12,14	3,23	37,16	198,97
79	6,43	0,26	0,32	50,54	9,27	2,09	0	22,81	52,26
80	6,2	0,16	0	86,17	6,63	8,54	1,91	19,11	52,31
81	12,79	0	0,14	55,74	7,12	5,32	0	36,25	72,29
82	2,45	0,14	0,21	161,88	14,92	19,26	3,15	15,5	36,23
83	4,08	0,14	0	234,48	7,66	39,76	3	30,21	84,56
84	7,06	0,33	0,26	32,78	6,81	4,05	0	44,41	85,70
85	3,58	0,61	0,14	128,26	10,1	2,1	2,5	15,27	48,18
86	3,52	0,52	0,14	207,54	8,2	17,83	3,02	16,19	61,55
87	1,66	1,34	0	229,53	1,7	5,43	2,31	39,06	155,84
88	5,76	0,16	0,34	59,36	6,86	3,34	0	20	44,50
89	3,29	2,53	0	286,82	8,15	6,79	5,15	19,06	61,57
90	4,95	0,13	0	68,43	1,77	6,85	0	18,77	62,47
91	6,54	1,52	0,15	20,75	2,86	2,15	2,96	29,86	86,49
92	3,27	0,09	0	60,23	3,25	4,13	0	13,89	51,88
93	6,08	0,37	0,72	26,57	6,36	1,92	0	18,89	60,90
94	2,64	0,3	0,53	10,62	3,74	1,11	0	11,1	34,78
95	16,32	0,3	0,34	29,45	4,9	2,79	0	47,33	57,44
96	18,4	0,23	0,14	31,83	4,64	6,66	1,02	58,2	70,06
97	5,48	0,33	0,21	354,6	9,24	22,89	2,36	17,02	64,33

ID	Longitude	Latitude	Soil Order	Microclimate	Slope	Soil Cover	Height	Sand	Clay
98	-76,133217	3,45497166	Andisoles	Cool (18-12 °C)	ST	FMFCP	2156	28,21	37,36
99	-76,424668	3,497497165	Mollisols	Hot (>24 °C)	Fl	SC	948	47,03	46,63
100	-76,226583	3,343897927	Alfisols	Hot (>24 °C)	SI	SC	1053	43,23	21,88
101	-76,258487	3,340275537	Mollisols	Hot (>24 °C)	Fl	SC	1017	63,98	11,54
102	-76,223864	3,339013976	Mollisols	Hot (>24 °C)	SI	SC	1042	45,51	17,7
103	-76,231719	3,303773232	Mollisols	Hot (>24 °C)	SI	SC	1571	62,71	10,89
104	-76,195102	3,284131836	Mollisols	Temperate or Medium (24-18 °C)	Fl	MA	1423	41,12	33,05
105	-76,426619	3,533867207	Vertisols	Hot (>24 °C)	Fl	SC	947	16,72	41,73
106	-76,351386	3,402744435	Mollisols	Hot (>24 °C)	Fl	SC	974	58,49	33,24
107	-76,240518	3,49668781	Alfisols	Hot (>24 °C)	SI	CP	1039	36,26	25,06
108	-76,269343	3,419484481	Mollisols	Hot (>24 °C)	Fl	SC	1015	31,78	11,47
109	-76,136799	3,444135569	Andisoles	Cool (18-12 °C)	ST	STV	2276	21,94	49,36
110	-76,227358	3,380750831	Alfisols	Hot (>24 °C)	Fl	SC	1064	34,03	32,31
111	-76,245251	3,43708001	Vertisols	Hot (>24 °C)	SI	SC	1039	22,15	29,41
112	-76,240679	3,392974564	Mollisols	Hot (>24 °C)	Fl	SC	1054	6,63	61,15
113	-76,253162	3,377241135	Mollisols	Hot (>24 °C)	Fl	SC	1034	8,82	55,91
114	-76,258846	3,364560052	Alfisols	Hot (>24 °C)	Fl	SC	1020	25,87	36,39
115	-76,209203	3,406269776	Mollisols	Hot (>24 °C)	SI	MCP	1159	20,74	35,87
116	-76,3451	3,444616666	Mollisols	Hot (>24 °C)	Fl	SC	970	45,38	32,44
117	-76,433053	3,509207941	Mollisols	Hot (>24 °C)	Fl	CP	948	77,5	12,36
118	-76,433843	3,413341583	Vertisols	Hot (>24 °C)	Fl	SC	951	15,78	52,92
119	-76,238616	3,40856824	Mollisols	Hot (>24 °C)	SI	SC	1066	34,35	12,9
120	-76,113448	3,457216684	Andisoles	Cool (18-12 °C)	ST	STV	2431	49,12	16,75
121	-76,220559	3,41141794	Mollisols	Hot (>24 °C)	SI	CP	1118	27,59	29,41
122	-76,353769	3,38899252	Mollisols	Hot (>24 °C)	Fl	SC	973	75,7	12,24
123	-76,354601	3,34490681	Mollisols	Hot (>24 °C)	Fl	SC	979	63,38	18,4
124	-76,240021	3,507820846	Vertisols	Hot (>24 °C)	SI	CPA	1050	44,38	19,03
125	-76,23022	3,391403157	Vertisols	Hot (>24 °C)	Fl	SC	1070	25,35	27,66
126	-76,162449	3,422611707	Mollisols	Temperate or Medium (24-18 °C)	SI	CPW	1523	24,68	35
127	-76,235651	3,517044611	Alfisols	Hot (>24 °C)	SI	SC	1049	36,65	26,16
128	-76,238252	3,40159122	Mollisols	Hot (>24 °C)	SI	OAC	943	32,85	27,72
129	-76,231821	3,415639985	Mollisols	Hot (>24 °C)	SI	SC	1086	27,97	35,36
130	-76,281221	3,347906647	Mollisols	Hot (>24 °C)	Fl	SC	1008	23,49	33,5

ID	Silt	TEXTURE	DA	pH	CE	MO	CO	Total Nitrogen	S	B	P	Ca
98	34,43	CL	1,49	5,96	0,63	9,07	5,26	0,62	9,6	0,33	58,33	18,42
99	6,34	SC	1,40	7,35	0,28	1,57	0,91	0,15	5,28	0,24	20,32	35,19
100	34,89	L	1,47	6,49	0,13	2,28	1,32	0,14	3,74	0,2	13,26	9,06
101	24,48	SL	1,53	6,23	0,12	5,14	2,98	0,16	3,55	0,17	54,45	8,6
102	36,79	L	1,63	6,37	0,22	2,19	1,27	0,16	5,74	0,17	32,87	8,05
103	26,4	SL	1,44	6,16	0,12	1,59	0,92	0,11	5,04	0,21	3,14	6,3
104	25,83	CL	1,39	6,61	0,13	2,17	1,26	0,21	5,31	0,2	0	27,6
105	41,55	SiC	1,43	8,86	1,14	0,83	0,48	0,14	30,88	0,17	20,92	30,28
106	8,27	SCL	1,52	7,2	0,36	2,78	1,51	0,16	6,8	0,21	28,24	15,58
107	38,68	L	0,96	6,67	0,24	4,95	2,87	0,36	5,13	0,2	7,86	23,73
108	56,75	SiL	1,66	8,46	0,64	1,86	1,08	0,14	3,69	0,45	23,48	14,93
109	28,7	C	0,97	5,62	0,9	13,65	7,92	0,89	13,77	0,39	17,34	8,54
110	33,66	CL	1,41	7,14	0,12	2,02	1,17	0,15	3,72	0,22	1,68	16,55
111	48,44	CL	1,17	7,05	0,24	2,98	1,73	0,2	15,49	0,32	31,09	28,72
112	32,22	C	1,69	6,75	0,21	2,74	1,59	0,2	4,82	0,28	98,75	12,94
113	35,27	C	1,54	6,82	0,15	2,38	1,38	0,17	3,87	0,25	13,7	9,88
114	37,74	CL	1,39	7,08	0,13	2,53	1,47	0,17	3,52	0,38	5,57	17,07
115	43,39	CL	1,36	6,92	0,17	3,59	2,08	0,21	3,29	0,22	20,26	13,92
116	22,18	SCL	1,51	6,97	0,23	2,16	1,25	0,1	5,85	0,47	113	8,46
117	10,14	SL	1,48	8,35	10,28	0,79	0,46	0,08	359,71	0,12	28,63	35,33
118	31,3	C	1,42	7,35	0,16	2,74	1,59	0,18	4,61	0,26	9,68	14,66
119	52,75	SiL	1,73	6,02	0,22	2,59	1,5	0,14	9,89	0,32	41,1	7,7
120	34,13	L	1,40	6,45	1,24	13,34	7,74	0,77	22,07	0,93	917,77	36,88
121	43	CL	1,40	7,13	0,63	3,1	1,8	0,21	12,16	0,26	221,18	11,74
122	12,06	SL	1,50	7,6	0,13	1,69	0,98	0,1	2,05	0,19	22,87	4,83
123	18,22	SL	1,40	6,97	0,29	1,64	0,95	0,11	16,26	0,21	16,8	10,28
124	36,59	L	0,99	6,87	0,22	3,59	2,08	0,29	7,39	0,15	16,26	24
125	46,99	CL	1,47	6,89	0,2	1,88	1,09	0,13	8,43	0,4	5,91	11,09
126	40,32	CL	1,01	6,47	0,43	5,76	3,34	0,45	7,68	0,26	8,41	13,18
127	37,19	L	1,33	7,06	0,4	2	1,16	0,15	24,44	0,46	41,69	12,3
128	39,43	CL	1,44	6,71	0,21	2,19	1,27	0,17	5,14	0,42	60,77	10,56
129	36,67	CL	1,59	6,25	0,12	1,9	1,1	0,14	5,59	0,35	41,96	7,12
130	43,01	CL	1,63	7,01	0,2	3,02	1,75	0,17	3,08	0,19	52,62	10,11

ID	Mg	K	Na	Fe	Cu	Mn	Zn	CICA	COS
98	3,48	3,55	0	206,64	2,01	4,14	4,3	35,57	235,24
99	10,03	0,38	0,56	26,22	9,32	3,51	0	33,22	38,22
100	4,23	0,17	0	93,3	4,47	9,46	0	16,53	58,25
101	1,63	0,15	0	110,58	1,47	2,83	1,59	15,29	136,79
102	3,5	0,35	0	153,87	5,71	9,05	1,28	16,24	61,96
103	2,66	0,1	0	91,27	1,03	11,61	0	9,54	39,78
104	17,15	0,16	0,2	28,11	2,55	7,17	0	46,68	52,45
105	13,61	0,47	3,14	9,85	2,31	1,56	0	22,36	20,59
106	8,7	0,31	0,33	48,67	6,03	2,18	0	21,32	68,88
107	13,06	0,17	0,33	45,93	6,7	8,14	0	42,42	83,01
108	3,26	0,09	0,29	42,73	6,49	3,22	0	10,06	53,84
109	5,13	3,62	0	240,37	1,45	4,08	2,73	34,62	230,94
110	11,13	0,22	0,23	21,02	4,23	2,13	0	31,13	49,49
111	20,8	0,61	0,31	17,91	4,89	2,68	0	49,32	60,92
112	4,16	0,29	0	98,41	6,51	2,83	2,2	21,38	80,78
113	4,57	0,18	0,16	92,58	5,85	5,79	1,05	17,68	63,72
114	12,62	0,17	0,18	43,76	6,43	5,82	0	29,78	61,50
115	2,42	0,17	0	99,26	5,95	1,19	1,46	17,93	84,92
116	2,06	0,51	0	58,16	5,01	2,43	2,01	11,58	56,76
117	12,37	0,23	7,79	21,34	2,38	5,38	0	6,65	20,42
118	7,06	0,2	0,35	64,62	5,23	1,56	0	21,85	67,93
119	1,64	0,27	0	155,67	15,76	8,54	11	10,96	77,64
120	4,46	3,18	0,15	44,14	8,33	2,84	27,38	53,15	324,80
121	1,96	1,29	0	236,36	9,48	3,33	9,95	15,77	75,54
122	1,34	0	0	15,3	0	1,51	0	6,27	44,16
123	2,86	0,19	0,15	48,58	2,49	2	0	11,48	39,80
124	17,56	0,3	0,25	30,01	9,38	9,14	3,14	41,62	61,78
125	3,29	0,15	0,15	66,81	3,4	1,96	0	18,11	48,07
126	1,55	0,24	0	80,42	2,31	1,43	3,02	21,83	101,20
127	2,2	0,19	0	93,5	8	3,38	4,14	17,06	46,24
128	1,79	0,27	0	109,15	12,74	2,98	6,11	15,43	55,05
129	1,36	0,15	0,14	114,65	15,06	3,71	5,12	12,75	52,47
130	3,43	0,18	0,19	111,86	2,63	1,26	0	14,93	85,58
