# Supplementary material for: Soil organic carbon in agricultural soils of an inter-Andean valley in Colombia: understanding the effects of environmental and geographic variables
Source: Environ Monit Assess. 2025 May 30;197(6):697. doi: 10.1007/s10661-025-14123-1 (PMC12125077; doi:10.1007/s10661-025-14123-1)
Supplement: Supplementary file 2 — (PDF 90.9 KB) [file 10661_2025_14123_MOESM2_ESM.pdf]

Table A2.Cross-validation of ordinary univariate kriging, multivariate cokriging (SOC and NT) and multivariate cokriging (SOC, pH and Fe).

Table A21. Cross-validation of ordinary univariate kriging

FID	Shape	Measured	Predicted	Error	StdError	Stdd_Error	NormValue	Source_ID	Included
0	Point	91,233	72,473172	-18,759828	27,872484	-0,673059	-1,036433	0	Yes
1	Point	43,26869	143,672638	100,403948	69,933014	1,435716	2,665285	1	Yes
2	Point	125,862605	72,085799	-53,776806	35,202353	-1,527648	-1,574445	2	Yes
3	Point	81,554183	65,914982	-15,639201	32,107606	-0,487087	-0,801095	3	Yes
4	Point	46,984551	68,071864	21,087312	33,63511	0,626943	0,723731	4	Yes
5	Point	64,283653	68,16512	3,881467	33,281046	0,116627	-0,009641	5	Yes
6	Point	67,431006	57,713086	-9,71792	28,178116	-0,344875	-0,67449	6	Yes
7	Point	34,562142	66,316087	31,753944	32,378006	0,980726	1,399916	7	Yes
8	Point	56,663285	58,10269	1,439405	28,367549	0,050741	-0,086877	8	Yes
9	Point	57,265937	67,943575	10,677638	33,172583	0,321881	0,283331	9	Yes
10	Point	60,31018	59,710398	-0,599782	29,153714	-0,020573	-0,223589	10	Yes
11	Point	55,317266	82,118417	26,801152	40,096723	0,668413	0,774794	11	Yes
12	Point	91,267719	129,144071	37,876351	66,234122	0,571856	0,626832	12	Yes
13	Point	69,591116	68,350605	-1,240512	34,311197	-0,036155	-0,263315	13	Yes
14	Point	78,689194	85,561339	6,872146	41,777307	0,164495	0,048223	14	Yes
15	Point	59,648646	58,160593	-1,488053	28,395762	-0,052404	-0,303461	15	Yes
16	Point	41,013501	62,574761	21,56126	30,228064	0,713286	0,88358	16	Yes
17	Point	74,722275	59,405729	-15,316546	29,004154	-0,528081	-0,827961	17	Yes
18	Point	38,928998	72,189692	33,260694	38,235635	0,869887	1,104836	18	Yes
19	Point	61,56	39,42496	-22,13504	19,247571	-1,150017	-1,399916	19	Yes
20	Point	55,699487	52,580645	-3,118842	26,394717	-0,118162	-0,427204	20	Yes
21	Point	64,548057	53,962874	-10,585183	26,346954	-0,401761	-0,698899	21	Yes
22	Point	43,032	41,829617	-1,202383	20,412282	-0,058905	-0,323715	22	Yes
23	Point	72,367624	141,106721	68,739097	68,86063	0,998235	1,453252	23	Yes
24	Point	71,794229	62,887924	-8,906305	30,703017	-0,290079	-0,580558	24	Yes
25	Point	29,295	38,311775	9,016775	19,650058	0,458868	0,491494	25	Yes
26	Point	62,750602	57,011667	-5,738935	27,835801	-0,206171	-0,51337	26	Yes
27	Point	49,353832	65,914972	16,56114	32,182599	0,514599	0,580558	27	Yes

Table A2.Cross-validation of ordinary univariate kriging, multivariate cokriging (SOC and NT) and multivariate cokriging (SOC, pH and Fe).

Table A21. Cross-validation of ordinary univariate kriging

FID	Shape	Measured	Predicted	Error	StdError	Stdd_Error	NormValue	Source_ID	Included
28	Point	62,838555	68,657655	5,819101	33,527289	0,173563	0,086877	28	Yes
29	Point	63,460233	67,268782	3,808549	32,840364	0,115972	-0,028927	29	Yes
30	Point	47,657261	71,066043	23,408782	34,695829	0,674686	0,801095	30	Yes
31	Point	112,834225	61,213017	-51,621208	29,887368	-1,727191	-1,644854	31	Yes
32	Point	55,758743	61,839152	6,08041	30,192515	0,201388	0,125661	32	Yes
33	Point	83,952246	85,483775	1,53153	12,704304	0,120552	0,009641	33	Yes
34	Point	35,32575	42,999592	7,673843	14,254666	0,538339	0,603533	34	Yes
35	Point	57,991549	66,599436	8,607887	32,514472	0,26474	0,203862	35	Yes
36	Point	54,258181	82,509046	28,250866	40,279545	0,70137	0,827961	36	Yes
37	Point	46,549305	81,364164	34,81486	37,877292	0,919149	1,259936	37	Yes
38	Point	92,033796	72,931529	-19,102267	35,285062	-0,54137	-0,85544	38	Yes
39	Point	51,36	81,095473	29,735473	37,602691	0,79078	1,003988	39	Yes
40	Point	37,038149	42,363924	5,325775	14,367813	0,370674	0,38532	40	Yes
41	Point	82,008	63,554717	-18,453283	31,105346	-0,593251	-0,942076	41	Yes
42	Point	181,024165	123,829121	-57,195045	60,293823	-0,948605	-1,259936	42	Yes
43	Point	50,628395	119,659945	69,03155	58,445393	1,181129	1,928072	43	Yes
44	Point	85,488	75,915108	-9,572892	37,930408	-0,25238	-0,557885	44	Yes
45	Point	90,576	74,581722	-15,994278	34,006117	-0,470335	-0,774794	45	Yes
46	Point	63,441	62,839274	-0,601726	30,318505	-0,019847	-0,203862	46	Yes
47	Point	134,508491	133,574757	-0,933734	65,942652	-0,01416	-0,184213	47	Yes
48	Point	124,121467	106,777273	-17,344193	52,158635	-0,332528	-0,626832	48	Yes
49	Point	77,172	153,134809	75,962809	74,395003	1,021074	1,574445	49	Yes
50	Point	51,681	77,534936	25,853936	28,244205	0,915371	1,218387	50	Yes
51	Point	127,293882	138,871756	11,577875	69,692183	0,166129	0,067537	51	Yes
52	Point	106,997344	119,248714	12,251369	57,907035	0,21157	0,164636	52	Yes
53	Point	81,51	57,185866	-24,324134	20,928423	-1,162254	-1,453252	53	Yes
54	Point	68,704575	69,318546	0,613971	20,63859	0,029749	-0,106249	54	Yes
55	Point	110,524892	75,535161	-34,989731	38,066955	-0,919163	-1,218387	55	Yes

Table A2.Cross-validation of ordinary univariate kriging, multivariate cokriging (SOC and NT) and multivariate cokriging (SOC, pH and Fe).

Table A21. Cross-validation of ordinary univariate kriging

FID	Shape	Measured	Predicted	Error	StdError	Stdd_Error	NormValue	Source_ID	Included
56	Point	91,259023	68,63444	-22,624583	33,522338	-0,674911	-1,070009	56	Yes
57	Point	99,406871	86,531125	-12,875747	42,248129	-0,304765	-0,603533	57	Yes
58	Point	70,016959	80,588144	10,571185	38,463626	0,274836	0,223589	58	Yes
59	Point	111,222	88,694897	-22,527103	40,573525	-0,555217	-0,912437	59	Yes
60	Point	108,166221	79,941642	-28,224579	40,128517	-0,703355	-1,104836	60	Yes
61	Point	49,92	55,684691	5,764691	28,381179	0,203117	0,145121	61	Yes
62	Point	69,657	79,949226	10,292226	36,782944	0,27981	0,243404	62	Yes
63	Point	97,644645	83,615252	-14,029393	40,798838	-0,343867	-0,650476	63	Yes
64	Point	38,648897	82,169508	43,520611	40,099623	1,085312	1,724512	64	Yes
65	Point	48,357634	57,700898	9,343264	28,827897	0,324105	0,303461	65	Yes
66	Point	50,6463	89,26378	38,61748	44,079526	0,876087	1,141057	66	Yes
67	Point	122,588941	85,06107	-37,527871	38,747331	-0,968528	-1,303783	67	Yes
68	Point	81,708123	90,523015	8,814891	44,193423	0,199462	0,106249	68	Yes
69	Point	76,221113	91,93146	15,710347	44,8879	0,349991	0,344102	69	Yes
70	Point	40,92961	74,174146	33,244535	34,96354	0,950834	1,303783	70	Yes
71	Point	48,110696	79,943202	31,832506	39,059784	0,814969	1,070009	71	Yes
72	Point	80,218455	75,900716	-4,317739	37,483734	-0,11519	-0,406173	72	Yes
73	Point	63,090744	118,076995	54,986251	57,650352	0,953789	1,35029	73	Yes
74	Point	108,672	68,378481	-40,293519	21,42202	-1,880939	-1,928072	74	Yes
75	Point	36,465569	71,43292	34,967351	24,968445	1,400462	2,272159	75	Yes
76	Point	93,024903	89,333847	-3,691056	44,848741	-0,0823	-0,364634	76	Yes
77	Point	198,974713	68,37868	-130,596033	33,385518	-3,911757	-2,665285	77	Yes
78	Point	52,26	38,768132	-13,491868	19,153173	-0,704419	-1,141057	78	Yes
79	Point	52,308603	56,421277	4,112673	28,32272	0,145208	0,028927	79	Yes
80	Point	72,289468	72,295863	0,006396	35,280596	0,000181	-0,164636	80	Yes
81	Point	36,225	53,891709	17,666709	27,0546	0,653002	0,749018	81	Yes
82	Point	84,563598	77,498823	-7,064775	39,084476	-0,180757	-0,491494	82	Yes
83	Point	85,701992	86,382675	0,680683	42,649267	0,01596	-0,145121	83	Yes

Table A2.Cross-validation of ordinary univariate kriging, multivariate cokriging (SOC and NT) and multivariate cokriging (SOC, pH and Fe).

Table A21. Cross-validation of ordinary univariate kriging

FID	Shape	Measured	Predicted	Error	StdError	Stdd_Error	NormValue	Source_ID	Included
84	Point	48,183995	96,522922	48,338928	47,129716	1,025657	1,644854	84	Yes
85	Point	61,548057	56,544427	-5,003629	28,382496	-0,176293	-0,448425	85	Yes
86	Point	155,839761	151,687548	-4,152213	80,316657	-0,051698	-0,283331	86	Yes
87	Point	44,496	32,031076	-12,464924	16,965269	-0,734732	-1,178841	87	Yes
88	Point	61,569152	60,85188	-0,717272	31,215611	-0,022978	-0,243404	88	Yes
89	Point	62,472	78,442063	15,970063	38,206469	0,417994	0,427204	89	Yes
90	Point	86,487	59,539953	-26,947047	23,553653	-1,144071	-1,35029	90	Yes
91	Point	51,878024	82,664993	30,786969	42,533045	0,723836	0,912437	91	Yes
92	Point	60,9	36,601967	-24,298033	18,374172	-1,322402	-1,511075	92	Yes
93	Point	34,776	48,441465	13,665465	23,651191	0,577792	0,650476	93	Yes
94	Point	57,442903	53,932321	-3,510582	19,694506	-0,178252	-0,469851	94	Yes
95	Point	70,064069	88,758648	18,694578	43,308797	0,431658	0,448425	95	Yes
96	Point	64,329217	76,287081	11,957864	38,293715	0,312267	0,263315	96	Yes
97	Point	235,240005	123,068743	-112,171262	61,831481	-1,814145	-1,816911	97	Yes
98	Point	38,22	45,836129	7,616129	22,380633	0,3403	0,323715	98	Yes
99	Point	58,252855	76,005446	17,752591	37,016324	0,479588	0,557885	99	Yes
100	Point	136,787589	70,513134	-66,274454	34,440152	-1,924337	-2,069902	100	Yes
101	Point	61,956794	76,205365	14,248572	37,152071	0,38352	0,406173	101	Yes
102	Point	39,781208	69,885002	30,103793	34,014812	0,88502	1,178841	102	Yes
103	Point	52,448335	85,216888	32,768553	23,982422	1,366357	2,069902	103	Yes
104	Point	20,592	40,990106	20,398106	20,015108	1,019135	1,511075	104	Yes
105	Point	68,882695	57,068376	-11,814319	27,862695	-0,424019	-0,749018	105	Yes
106	Point	83,014127	63,910338	-19,103789	31,203849	-0,612225	-0,972566	106	Yes
107	Point	53,843669	69,885608	16,041939	34,121469	0,470142	0,535495	107	Yes
108	Point	230,937963	125,130239	-105,807725	60,786392	-1,740648	-1,724512	108	Yes
109	Point	49,491	70,288202	20,797202	34,329672	0,605808	0,67449	109	Yes
110	Point	60,918401	73,713153	12,794752	36,407141	0,351435	0,364634	110	Yes
111	Point	80,784958	63,750063	-17,034894	31,119156	-0,547409	-0,88358	111	Yes

Table A2.Cross-validation of ordinary univariate kriging, multivariate cokriging (SOC and NT) and multivariate cokriging (SOC, pH and Fe).

Table A21. Cross-validation of ordinary univariate kriging

FID	Shape	Measured	Predicted	Error	StdError	Stdd_Error	NormValue	Source_ID	Included
112	Point	63,721113	60,421988	-3,299125	29,500577	-0,111833	-0,38532	112	Yes
113	Point	61,499784	78,800609	17,300825	38,474334	0,449672	0,469851	113	Yes
114	Point	84,922661	84,143547	-0,779114	12,505128	-0,062304	-0,344102	114	Yes
115	Point	56,763325	59,240534	2,477209	28,923474	0,085647	-0,048223	115	Yes
116	Point	20,424	43,523692	23,099692	21,238838	1,087616	1,816911	116	Yes
117	Point	67,93493	56,555807	-11,379123	27,612682	-0,412098	-0,723731	117	Yes
118	Point	77,644234	69,600653	-8,043581	33,949985	-0,236924	-0,535495	118	Yes
119	Point	324,802711	135,260309	-189,542402	67,879	-2,792357	-2,272159	119	Yes
120	Point	75,535514	76,239738	0,704223	37,655025	0,018702	-0,125661	120	Yes
121	Point	44,158762	71,478725	27,319963	34,895206	0,782915	0,942076	121	Yes
122	Point	39,802569	65,771319	25,96875	32,110454	0,808732	1,036433	122	Yes
123	Point	61,776	69,695342	7,919342	34,026359	0,232741	0,184213	123	Yes
124	Point	48,069	73,330142	25,261142	35,804606	0,705528	0,85544	124	Yes
125	Point	101,202	104,050517	2,848517	50,797748	0,056076	-0,067537	125	Yes
126	Point	46,239443	75,797891	29,558449	37,453604	0,789202	0,972566	126	Yes
127	Point	55,050457	71,154671	16,104213	34,704213	0,464042	0,51337	127	Yes
128	Point	52,47	74,682939	22,212939	36,463355	0,609185	0,698899	128	Yes
129	Point	85,575	65,843428	-19,731572	32,155346	-0,613633	-1,003988	129	Yes

Table A22. Cross-validation multivariate cokriging (SOC and NT)

FID	Shape	Measured	Predicted	Error	StdError	Stdd_Error	NormValue	Source_ID	Included
0	Point	91,233	98,042533	6,809533	33,645355	0,202391	0,048223	0	Yes
1	Point	43,26869	87,821464	44,552774	37,324533	1,193659	1,816911	1	Yes
2	Point	125,862605	74,203834	-51,65877	31,638634	-1,632775	-1,644854	2	Yes
3	Point	81,554183	68,504663	-13,04952	29,136237	-0,447879	-1,003988	3	Yes
4	Point	46,984551	62,159077	15,174526	26,811324	0,565975	0,801095	4	Yes
5	Point	64,283653	60,971231	-3,312422	25,991735	-0,127441	-0,406173	5	Yes
6	Point	67,431006	43,460736	-23,970271	18,527204	-1,293788	-1,511075	6	Yes
7	Point	34,562142	71,773007	37,210864	30,596204	1,216192	1,928072	7	Yes
8	Point	56,663285	63,921897	7,258612	27,248962	0,266381	0,164636	8	Yes
9	Point	57,265937	53,912938	-3,352999	22,982596	-0,145893	-0,448425	9	Yes
10	Point	60,31018	81,499444	21,189264	34,743609	0,609875	0,942076	10	Yes
11	Point	55,317266	58,687266	3,370001	25,020034	0,134692	-0,028927	11	Yes
12	Point	91,267719	170,843754	79,576035	76,049945	1,046365	1,574445	12	Yes
13	Point	69,591116	66,476321	-3,114796	29,012217	-0,107362	-0,364634	13	Yes
14	Point	78,689194	82,905565	4,216372	35,343953	0,119295	-0,067537	14	Yes
15	Point	59,648646	51,97132	-7,677326	22,154542	-0,346535	-0,85544	15	Yes
16	Point	41,013501	52,869833	11,856332	22,309776	0,531441	0,67449	16	Yes
17	Point	74,722275	60,444244	-14,278031	25,766888	-0,554123	-1,070009	17	Yes
18	Point	38,928998	67,952329	29,023331	31,449343	0,92286	1,399916	18	Yes
19	Point	61,56	46,911491	-14,648509	19,996625	-0,732549	-1,218387	19	Yes
20	Point	55,699487	52,363284	-3,336203	22,852742	-0,145987	-0,469851	20	Yes
21	Point	64,548057	48,407756	-16,1403	20,636	-0,782143	-1,303783	21	Yes
22	Point	43,032	33,603513	-9,428487	14,317358	-0,658535	-1,141057	22	Yes
23	Point	72,367624	69,15988	-3,207744	29,467367	-0,108857	-0,38532	23	Yes
24	Point	71,794229	61,529547	-10,264682	26,228383	-0,391358	-0,942076	24	Yes
25	Point	29,295	33,113712	3,818712	14,74128	0,259049	0,145121	25	Yes
26	Point	62,750602	58,087753	-4,662849	24,762845	-0,1883	-0,557885	26	Yes
27	Point	49,353832	59,247183	9,89335	25,256929	0,391708	0,427204	27	Yes

Table A22. Cross-validation multivariate cokriging (SOC and NT)

FID	Shape	Measured	Predicted	Error	StdError	Stdd_Error	NormValue	Source_ID	Included
28	Point	62,838555	66,224813	3,386258	28,236308	0,119926	-0,048223	28	Yes
29	Point	63,460233	63,648175	0,187942	27,130176	0,006927	-0,263315	29	Yes
30	Point	47,657261	58,930949	11,273688	25,120727	0,44878	0,51337	30	Yes
31	Point	112,834225	102,883122	-9,951103	43,859467	-0,226886	-0,603533	31	Yes
32	Point	55,758743	67,52289	11,764147	28,78474	0,408694	0,469851	32	Yes
33	Point	83,952246	87,676476	3,72423	12,79022	0,291178	0,184213	33	Yes
34	Point	35,32575	37,249736	1,923986	11,301563	0,170241	0,028927	34	Yes
35	Point	57,991549	61,826449	3,8349	26,354282	0,145513	-0,009641	35	Yes
36	Point	54,258181	77,867446	23,609266	33,190275	0,711331	1,104836	36	Yes
37	Point	46,549305	86,056285	39,50698	35,088103	1,125937	1,644854	37	Yes
38	Point	92,033796	90,009491	-2,024305	38,019713	-0,053244	-0,344102	38	Yes
39	Point	51,36	73,065269	21,705269	29,693479	0,730978	1,141057	39	Yes
40	Point	37,038149	43,6033	6,565151	13,543033	0,484762	0,580558	40	Yes
41	Point	82,008	71,623846	-10,384154	30,602664	-0,339322	-0,801095	41	Yes
42	Point	181,024165	186,717397	5,693231	79,380887	0,07172	-0,164636	42	Yes
43	Point	50,628395	78,495695	27,8673	33,474411	0,832496	1,35029	43	Yes
44	Point	85,488	77,964677	-7,523323	34,000107	-0,221273	-0,580558	44	Yes
45	Point	90,576	89,28127	-1,29473	35,725781	-0,036241	-0,323715	45	Yes
46	Point	63,441	63,970676	0,529676	26,959905	0,019647	-0,243404	46	Yes
47	Point	134,508491	151,348219	16,839728	65,222293	0,25819	0,125661	47	Yes
48	Point	124,121467	104,276501	-19,844966	44,473923	-0,446216	-0,972566	48	Yes
49	Point	77,172	85,943042	8,771042	36,458984	0,240573	0,106249	49	Yes
50	Point	51,681	67,224844	15,543844	22,131212	0,702349	1,070009	50	Yes
51	Point	127,293882	169,468958	42,175076	73,939317	0,570401	0,827961	51	Yes
52	Point	106,997344	94,272531	-12,724813	39,973838	-0,318329	-0,774794	52	Yes
53	Point	81,51	72,6385	-8,8715	24,035914	-0,369094	-0,912437	53	Yes
54	Point	68,704575	63,745197	-4,959377	17,433239	-0,284478	-0,723731	54	Yes
55	Point	110,524892	61,005158	-49,519734	26,794928	-1,848101	-1,928072	55	Yes

Table A22. Cross-validation multivariate cokriging (SOC and NT)

FID	Shape	Measured	Predicted	Error	StdError	Stdd_Error	NormValue	Source_ID	Included
56	Point	91,259023	82,903028	-8,355995	35,353923	-0,236353	-0,626832	56	Yes
57	Point	99,406871	115,324868	15,917997	49,162453	0,323784	0,263315	57	Yes
58	Point	70,016959	70,164738	0,147779	29,269006	0,005049	-0,283331	58	Yes
59	Point	111,222	113,689605	2,467605	45,765993	0,053918	-0,184213	59	Yes
60	Point	108,166221	84,011899	-24,154322	36,664035	-0,658802	-1,178841	60	Yes
61	Point	49,92	46,249241	-3,670759	20,543207	-0,178685	-0,51337	61	Yes
62	Point	69,657	82,613902	12,956902	33,332112	0,388721	0,406173	62	Yes
63	Point	97,644645	98,81676	1,172115	42,098102	0,027842	-0,203862	63	Yes
64	Point	38,648897	40,307997	1,6591	17,174763	0,096601	-0,125661	64	Yes
65	Point	48,357634	66,056495	17,698861	28,77716	0,615032	0,972566	65	Yes
66	Point	50,6463	64,915317	14,269017	27,965172	0,510242	0,603533	66	Yes
67	Point	122,588941	93,028826	-29,560115	37,298768	-0,792523	-1,35029	67	Yes
68	Point	81,708123	76,760338	-4,947785	32,719645	-0,151218	-0,491494	68	Yes
69	Point	76,221113	102,120815	25,899702	43,536229	0,5949	0,88358	69	Yes
70	Point	40,92961	70,89866	29,96905	29,237723	1,025013	1,511075	70	Yes
71	Point	48,110696	104,813561	56,702865	44,713469	1,268138	2,272159	71	Yes
72	Point	80,218455	84,208977	3,990522	36,279577	0,109994	-0,086877	72	Yes
73	Point	63,090744	76,677615	13,586871	32,687444	0,41566	0,491494	73	Yes
74	Point	108,672	62,612241	-46,059759	17,904351	-2,572546	-2,665285	74	Yes
75	Point	36,465569	56,965651	20,500082	18,028309	1,137105	1,724512	75	Yes
76	Point	93,024903	108,211345	15,186442	47,230879	0,321536	0,243404	76	Yes
77	Point	198,974713	115,889088	-83,085625	49,403284	-1,681783	-1,816911	77	Yes
78	Point	52,26	37,469768	-14,790232	16,159668	-0,915256	-1,399916	78	Yes
79	Point	52,308603	54,848042	2,539438	24,032343	0,105668	-0,106249	79	Yes
80	Point	72,289468	63,829009	-8,460459	27,196288	-0,311089	-0,749018	80	Yes
81	Point	36,225	49,111774	12,886774	21,520215	0,598822	0,912437	81	Yes
82	Point	84,563598	85,437896	0,874298	37,606525	0,023249	-0,223589	82	Yes
83	Point	85,701992	101,199126	15,497134	43,587954	0,355537	0,38532	83	Yes

Table A22. Cross-validation multivariate cokriging (SOC and NT)

FID	Shape	Measured	Predicted	Error	StdError	Stdd_Error	NormValue	Source_ID	Included
84	Point	48,183995	84,112096	35,928101	35,858372	1,001945	1,453252	84	Yes
85	Point	61,548057	72,128366	10,580309	31,601445	0,334805	0,344102	85	Yes
86	Point	155,839761	120,950066	-34,889695	55,446638	-0,629248	-1,104836	86	Yes
87	Point	44,496	41,82726	-2,66874	19,180466	-0,139138	-0,427204	87	Yes
88	Point	61,569152	72,257231	10,688079	32,358321	0,330304	0,283331	88	Yes
89	Point	62,472	80,683184	18,211184	34,308633	0,530805	0,650476	89	Yes
90	Point	86,487	54,666435	-31,820565	19,226869	-1,655005	-1,724512	90	Yes
91	Point	51,878024	57,153296	5,275272	25,625123	0,205863	0,067537	91	Yes
92	Point	60,9	36,3818	-24,5182	15,878442	-1,544119	-1,574445	92	Yes
93	Point	34,776	52,91352	18,13752	22,556813	0,804082	1,303783	93	Yes
94	Point	57,442903	70,006572	12,56367	23,069056	0,544611	0,723731	94	Yes
95	Point	70,064069	102,121477	32,057408	43,50632	0,736845	1,178841	95	Yes
96	Point	64,329217	74,409553	10,080337	32,602173	0,309192	0,223589	96	Yes
97	Point	235,240005	152,703962	-82,536043	66,695655	-1,237503	-1,453252	97	Yes
98	Point	38,22	47,486815	9,266815	20,244511	0,457745	0,535495	98	Yes
99	Point	58,252855	60,551388	2,298533	25,749002	0,089267	-0,145121	99	Yes
100	Point	136,787589	71,29117	-65,496419	30,402214	-2,154331	-2,069902	100	Yes
101	Point	61,956794	67,948602	5,991808	28,923412	0,207161	0,086877	101	Yes
102	Point	39,781208	52,876073	13,094865	22,470388	0,582761	0,85544	102	Yes
103	Point	52,448335	88,497066	36,048731	23,022697	1,565791	2,665285	103	Yes
104	Point	20,592	44,79688	24,20488	19,098633	1,267362	2,069902	104	Yes
105	Point	68,882695	61,988604	-6,894091	26,424935	-0,260893	-0,67449	105	Yes
106	Point	83,014127	95,612302	12,598175	40,759017	0,309089	0,203862	106	Yes
107	Point	53,843669	63,067517	9,223848	26,885728	0,343076	0,364634	107	Yes
108	Point	230,937963	201,583759	-29,354205	85,511212	-0,343279	-0,827961	108	Yes
109	Point	49,491	61,620788	12,129788	26,277649	0,461601	0,557885	109	Yes
110	Point	60,918401	80,204254	19,285853	34,558189	0,558069	0,774794	110	Yes
111	Point	80,784958	75,006598	-5,778359	31,967285	-0,180759	-0,535495	111	Yes

Table A22. Cross-validation multivariate cokriging (SOC and NT)

FID	Shape	Measured	Predicted	Error	StdError	Stdd_Error	NormValue	Source_ID	Included
112	Point	63,721113	63,269965	-0,451149	26,971549	-0,016727	-0,303461	112	Yes
113	Point	61,499784	84,069555	22,569771	35,838987	0,629755	1,003988	113	Yes
114	Point	84,922661	81,778475	-3,144187	11,929827	-0,263557	-0,698899	114	Yes
115	Point	56,763325	47,644589	-9,118736	20,310481	-0,448967	-1,036433	115	Yes
116	Point	20,424	29,892727	9,468727	12,736223	0,743449	1,218387	116	Yes
117	Point	67,93493	61,345863	-6,589066	26,151214	-0,25196	-0,650476	117	Yes
118	Point	77,644234	59,060193	-18,584041	25,152991	-0,73884	-1,259936	118	Yes
119	Point	324,802711	162,011325	-162,791387	70,684989	-2,303055	-2,272159	119	Yes
120	Point	75,535514	81,071968	5,536453	34,932	0,158492	0,009641	120	Yes
121	Point	44,158762	51,399883	7,241121	21,909019	0,330509	0,303461	121	Yes
122	Point	39,802569	56,356687	16,554118	24,023088	0,689092	1,036433	122	Yes
123	Point	61,776	91,747043	29,971043	39,108951	0,766347	1,259936	123	Yes
124	Point	48,069	58,104298	10,035298	24,770149	0,405137	0,448425	124	Yes
125	Point	101,202	130,157462	28,955462	55,480777	0,521901	0,626832	125	Yes
126	Point	46,239443	60,060287	13,820844	25,831933	0,535029	0,698899	126	Yes
127	Point	55,050457	71,756571	16,706114	30,556664	0,546726	0,749018	127	Yes
128	Point	52,47	61,106004	8,636004	26,049042	0,331529	0,323715	128	Yes
129	Point	85,575	73,999205	-11,575795	31,553112	-0,366867	-0,88358	129	Yes

Table A23. Cross-validation multivariate cokriging (SOC, pH and Fe).

FID	Shape	Measured	Predicted	Error	StdError	Stdd_Error	NormValue	Source_ID	Included
0	Point	91,233	70,676653	-20,556347	25,031146	-0,821231	-1,178841	0	Yes
1	Point	43,26869	124,341806	81,073115	63,481808	1,277108	2,069902	1	Yes
2	Point	125,862605	84,835417	-41,027188	43,312108	-0,947245	-1,35029	2	Yes
3	Point	81,554183	67,857534	-13,696649	34,644172	-0,395352	-0,85544	3	Yes
4	Point	46,984551	59,366917	12,382366	30,687106	0,403504	0,344102	4	Yes
5	Point	64,283653	64,344873	0,06122	32,850809	0,001864	-0,243404	5	Yes
6	Point	67,431006	62,90672	-4,524287	32,11657	-0,140871	-0,448425	6	Yes
7	Point	34,562142	68,329601	33,767458	34,885183	0,96796	1,453252	7	Yes
8	Point	56,663285	62,452475	5,789189	31,884659	0,181567	0,067537	8	Yes
9	Point	57,265937	61,897234	4,631297	31,601184	0,146555	-0,009641	9	Yes
10	Point	60,31018	60,81056	0,500379	31,046391	0,016117	-0,203862	10	Yes
11	Point	55,317266	78,877442	23,560176	40,270307	0,585051	0,698899	11	Yes
12	Point	91,267719	128,478769	37,21105	68,998851	0,5393	0,626832	12	Yes
Va	Point	69,591116	66,92501	-2,666107	35,147184	-0,075855	-0,344102	13	Yes
14	Point	78,689194	77,9732	-0,715994	39,808652	-0,017986	-0,323715	14	Yes
15	Point	59,648646	52,018416	-7,630229	26,557626	-0,287308	-0,626832	15	Yes
16	Point	41,013501	59,239557	18,226056	30,204383	0,603424	0,801095	16	Yes
17	Point	74,722275	52,334493	-22,387782	26,718996	-0,837898	-1,218387	17	Yes
18	Point	38,928998	61,724995	22,795996	34,293305	0,664736	0,912437	18	Yes
19	Point	61,56	36,817161	-24,742839	18,796735	-1,316337	-1,511075	19	Yes
20	Point	55,699487	49,878115	-5,821372	26,194622	-0,222235	-0,580558	20	Yes
21	Point	64,548057	56,789241	-7,758816	28,993336	-0,267607	-0,603533	21	Yes
22	Point	43,032	36,998537	-6,033463	18,889335	-0,319411	-0,723731	22	Yes
23	Point	72,367624	147,455829	75,088205	75,282505	0,997419	1,644854	23	Yes
24	Point	71,794229	65,640783	-6,153446	33,512426	-0,183617	-0,491494	24	Yes
25	Point	29,295	37,396209	8,101209	20,083439	0,403378	0,323715	25	Yes
26	Point	62,750602	54,498156	-8,252446	27,823639	-0,296598	-0,67449	26	Yes
27	Point	49,353832	70,67529	21,321458	36,082757	0,590904	0,723731	27	Yes

Table A23. Cross-validation multivariate cokriging (SOC, pH and Fe).

FID	Shape	Measured	Predicted	Error	StdError	Stdd_Error	NormValue	Source_ID	Included
28	Point	62,838555	79,840419	17,001864	40,761947	0,417101	0,406173	28	Yes
29	Point	63,460233	65,091123	1,63089	33,231801	0,049076	-0,125661	29	Yes
30	Point	47,657261	77,490076	29,832815	39,561997	0,754078	1,070009	30	Yes
31	Point	112,834225	67,141212	-45,693013	34,278459	-1,332995	-1,574445	31	Yes
32	Point	55,758743	72,234889	16,476146	36,878999	0,446762	0,491494	32	Yes
33	Point	83,952246	86,53276	2,580514	19,07297	0,135297	-0,028927	33	Yes
34	Point	35,32575	42,722099	7,396349	13,620364	0,543036	0,650476	34	Yes
35	Point	57,991549	66,251142	8,259593	33,82404	0,244193	0,125661	35	Yes
36	Point	54,258181	81,019466	26,761286	41,363902	0,646972	0,88358	36	Yes
37	Point	46,549305	82,720642	36,171338	40,14789	0,900952	1,399916	37	Yes
38	Point	92,033796	69,677826	-22,35597	36,063137	-0,619912	-1,003988	38	Yes
39	Point	51,36	72,426851	21,066851	35,245279	0,597721	0,749018	39	Yes
40	Point	37,038149	42,204344	5,166195	13,899817	0,371674	0,303461	40	Yes
41	Point	82,008	66,379311	-15,628689	33,895817	-0,46108	-0,942076	41	Yes
42	Point	181,024165	130,117365	-50,906801	66,430478	-0,766317	-1,104836	42	Yes
43	Point	50,628395	103,075699	52,447304	52,624552	0,996632	1,574445	43	Yes
44	Point	85,488	77,158104	-8,329896	40,860043	-0,203864	-0,557885	44	Yes
45	Point	90,576	94,672666	4,096666	44,302361	0,092471	-0,086877	45	Yes
46	Point	63,441	68,66428	5,22328	35,009751	0,149195	0,009641	46	Yes
47	Point	134,508491	126,216671	-8,29182	65,242133	-0,127093	-0,406173	47	Yes
48	Point	124,121467	116,127119	-7,994347	59,287859	-0,13484	-0,427204	48	Yes
49	Point	77,172	153,741583	76,569583	78,491652	0,975512	1,511075	49	Yes
50	Point	51,681	74,439677	22,758677	25,956353	0,876806	1,303783	50	Yes
51	Point	127,293882	147,66237	20,368488	77,548237	0,262656	0,164636	51	Yes
52	Point	106,997344	108,972078	1,974733	55,634905	0,035495	-0,145121	52	Yes
53	Point	81,51	60,308562	-21,201438	21,113668	-1,004157	-1,399916	53	Yes
54	Point	68,704575	74,900537	6,195963	20,805678	0,297802	0,203862	54	Yes
55	Point	110,524892	75,097786	-35,427106	40,226194	-0,880697	-1,259936	55	Yes

Table A23. Cross-validation multivariate cokriging (SOC, pH and Fe).

FID	Shape	Measured	Predicted	Error	StdError	Stdd_Error	NormValue	Source_ID	Included
56	Point	91,259023	62,537288	-28,721735	31,927959	-0,899579	-1,303783	56	Yes
57	Point	99,406871	85,716702	-13,69017	43,762041	-0,312832	-0,698899	57	Yes
58	Point	70,016959	74,627114	4,610156	37,72757	0,122196	-0,067537	58	Yes
59	Point	111,222	97,934549	-13,287451	44,926063	-0,295763	-0,650476	59	Yes
60	Point	108,166221	89,105817	-19,060404	46,795937	-0,407309	-0,88358	60	Yes
61	Point	49,92	58,918912	8,998912	31,507915	0,285608	0,184213	61	Yes
62	Point	69,657	70,105036	0,448036	33,360573	0,01343	-0,223589	62	Yes
63	Point	97,644645	90,333552	-7,311093	46,119141	-0,158526	-0,469851	63	Yes
64	Point	38,648897	88,715962	50,067065	45,293292	1,105397	1,928072	64	Yes
65	Point	48,357634	63,042203	14,684569	33,363059	0,440145	0,469851	65	Yes
66	Point	50,6463	73,429072	22,782771	37,914797	0,600894	0,774794	66	Yes
67	Point	122,588941	89,516923	-33,072018	40,69918	-0,812597	-1,141057	67	Yes
68	Point	81,708123	99,332983	17,62486	50,713735	0,347536	0,263315	68	Yes
69	Point	76,221113	107,57455	31,353436	54,921407	0,570878	0,67449	69	Yes
70	Point	40,92961	70,844084	29,914474	34,208198	0,874483	1,259936	70	Yes
71	Point	48,110696	73,907802	25,797106	37,733093	0,683673	0,972566	71	Yes
72	Point	80,218455	72,820473	-7,397982	37,60055	-0,196752	-0,51337	72	Yes
73	Point	63,090744	105,221913	42,131169	53,720286	0,784269	1,141057	73	Yes
74	Point	108,672	75,022494	-33,649506	21,838822	-1,540811	-1,724512	74	Yes
75	Point	36,465569	69,152023	32,686454	22,468021	1,454799	2,272159	75	Yes
76	Point	93,024903	88,371309	-4,653593	46,410194	-0,100271	-0,38532	76	Yes
77	Point	198,974713	77,544225	-121,430488	39,589642	-3,067229	-2,272159	77	Yes
78	Point	52,26	38,255561	-14,004439	19,774522	-0,708206	-1,070009	78	Yes
79	Point	52,308603	53,274473	0,965869	27,991711	0,034506	-0,164636	79	Yes
80	Point	72,289468	72,185577	-0,10389	36,853824	-0,002819	-0,263315	80	Yes
81	Point	36,225	66,760357	30,535357	35,077524	0,870511	1,178841	81	Yes
82	Point	84,563598	108,051931	23,488333	56,802297	0,41351	0,38532	82	Yes
83	Point	85,701992	88,398339	2,696347	45,644116	0,059073	-0,106249	83	Yes

Table A23. Cross-validation multivariate cokriging (SOC, pH and Fe).

FID	Shape	Measured	Predicted	Error	StdError	Stdd_Error	NormValue	Source_ID	Included
84	Point	48,183995	99,31219	51,128195	50,703119	1,008384	1,724512	84	Yes
85	Point	61,548057	69,014622	7,466565	36,26197	0,205906	0,106249	85	Yes
86	Point	155,839761	140,179232	-15,660529	77,794118	-0,201307	-0,535495	86	Yes
87	Point	44,496	35,80681	-8,68919	19,871411	-0,437271	-0,912437	87	Yes
88	Point	61,569152	67,526784	5,957632	36,290306	0,164166	0,048223	88	Yes
89	Point	62,472	79,222885	16,750885	40,518801	0,41341	0,364634	89	Yes
90	Point	86,487	49,679797	-36,807203	18,033447	-2,041052	-2,069902	90	Yes
91	Point	51,878024	78,002404	26,12438	42,332681	0,617121	0,85544	91	Yes
92	Point	60,9	38,775712	-22,124288	20,363943	-1,086444	-1,453252	92	Yes
93	Point	34,776	42,422738	7,646738	21,658622	0,353057	0,283331	93	Yes
94	Point	57,442903	51,575063	-5,867839	18,127174	-0,323704	-0,749018	94	Yes
95	Point	70,064069	89,441973	19,377903	45,663951	0,424359	0,427204	95	Yes
96	Point	64,329217	86,663419	22,334203	45,53508	0,490483	0,580558	96	Yes
97	Point	235,240005	128,028293	-107,211712	67,236957	-1,594535	-1,816911	97	Yes
98	Point	38,22	48,849296	10,629296	24,939654	0,426201	0,448425	98	Yes
99	Point	58,252855	76,675666	18,422811	39,146206	0,470615	0,535495	99	Yes
100	Point	136,787589	73,324599	-63,46299	37,435343	-1,695269	-1,928072	100	Yes
101	Point	61,956794	81,740777	19,783983	41,732534	0,474066	0,557885	101	Yes
102	Point	39,781208	72,061807	32,280599	36,797516	0,877249	1,35029	102	Yes
103	Point	52,448335	88,090045	35,64171	23,768012	1,499566	2,665285	103	Yes
104	Point	20,592	37,065616	16,473616	18,923582	0,870534	1,218387	104	Yes
105	Point	68,882695	58,433663	-10,449032	29,832883	-0,350252	-0,801095	105	Yes
106	Point	83,014127	65,34941	-17,664717	33,363668	-0,52946	-0,972566	106	Yes
107	Point	53,843669	63,533694	9,690025	32,436667	0,298737	0,223589	107	Yes
108	Point	230,937963	129,68003	-101,257934	66,2072	-1,52941	-1,644854	108	Yes
109	Point	49,491	64,259739	14,768739	32,807344	0,450166	0,51337	109	Yes
110	Point	60,918401	67,489599	6,571197	34,847975	0,188568	0,086877	110	Yes
111	Point	80,784958	68,81472	-11,970237	35,132857	-0,340713	-0,774794	111	Yes

Table A23. Cross-validation multivariate cokriging (SOC, pH and Fe).

FID	Shape	Measured	Predicted	Error	StdError	Stdd_Error	NormValue	Source_ID	Included
112	Point	63,721113	64,557513	0,836399	32,95937	0,025377	-0,184213	112	Yes
113	Point	61,499784	73,371152	11,871368	37,45911	0,316915	0,243404	113	Yes
114	Point	84,922661	84,843978	-0,078683	18,70074	-0,004207	-0,283331	114	Yes
115	Point	56,763325	65,396928	8,633602	33,387928	0,258585	0,145121	115	Yes
116	Point	20,424	42,74604	22,32204	21,823681	1,022836	1,816911	116	Yes
117	Point	67,93493	56,981244	-10,953685	29,091362	-0,376527	-0,827961	117	Yes
118	Point	77,644234	77,300134	-0,3441	39,465024	-0,008719	-0,303461	118	Yes
119	Point	324,802711	114,525945	-210,276766	60,145893	-3,496112	-2,665285	119	Yes
120	Point	75,535514	80,720657	5,185143	41,679777	0,124404	-0,048223	120	Yes
121	Point	44,158762	64,418246	20,259484	32,888269	0,616009	0,827961	121	Yes
122	Point	39,802569	65,464339	25,66177	33,422344	0,767803	1,104836	122	Yes
123	Point	61,776	67,389247	5,613247	34,405092	0,163152	0,028927	123	Yes
124	Point	48,069	74,610491	26,541491	38,091846	0,696776	1,003988	124	Yes
125	Point	101,202	97,376864	-3,825136	49,715052	-0,076941	-0,364634	125	Yes
126	Point	46,239443	75,394582	29,155139	38,958315	0,748368	1,036433	126	Yes
127	Point	55,050457	74,463728	19,41327	38,016917	0,510648	0,603533	127	Yes
128	Point	52,47	80,225026	27,755026	40,958306	0,677641	0,942076	128	Yes
129	Point	85,575	64,830019	-20,744981	33,098496	-0,626765	-1,036433	129	Yes
